# It’s complicated: Heterogeneous patterns of genetic structure in five fish species from a fragmented river suggest multiple processes can drive differentiation

**DOI:** 10.1111/eva.13268

**Published:** 2021-06-29

**Authors:** Rebecca R. Gehri, Kristen Gruenthal, Wesley A. Larson

**Affiliations:** ^1^ Wisconsin Cooperative Fishery Research Unit College of Natural Resources University of Wisconsin‐Stevens Point Stevens Point WI USA; ^2^ Office of Applied Science Wisconsin Department of Natural Resources College of Natural Resources University of Wisconsin‐Stevens Point Stevens Point WI USA; ^3^ Alaska Department of Fish and Game Gene Conservation Laboratory Juneau AK USA; ^4^ U.S. Geological Survey Wisconsin Cooperative Fishery Research Unit College of Natural Resources University of Wisconsin‐Stevens Point Stevens Point WI USA; ^5^ National Oceanographic and Atmospheric Administration National Marine Fisheries Service Alaska Fisheries Science Center Auke Bay Laboratories Juneau AK USA

**Keywords:** dams, fish passage, habitat fragmentation, Lake Michigan, population genomics, RADseq

## Abstract

Fragmentation of river systems by dams can have substantial genetic impacts on fish populations. However, genetic structure can exist naturally at small scales through processes other than isolation by physical barriers. We sampled individuals from five native fish species with varying life histories above and below a dam in the lower Boardman River, Michigan, USA, and used RADseq to investigate processes influencing genetic structure in this system. Species assessed were white sucker *Catostomus commersonii*, yellow perch *Perca flavescens*, walleye *Sander vitreus*, smallmouth bass *Micropterus dolomieu*, and rock bass *Ambloplites rupestris*. We detected significant differentiation within each species, but patterns of population structure varied substantially. Interestingly, genetic structure did not appear to be solely the result of fragmentation by the dam. While genetic structure in yellow perch and walleye generally coincided with “above dam” and “below dam” sampling locations, samples from our other three species did not. Specifically, samples from rock bass, smallmouth bass, and, to a much lesser extent, white sucker, aligned with a putative Great Lakes (GL) group that contained mostly individuals sampled below the dam and a putative Boardman River (BR) group that contained individuals sampled both above and below the dam, with some evidence of admixture among groups. We hypothesize that the GL and BR groups formed prior to dam construction and our samples largely represent a mixed stock that was sampled sympatrically outside of the spawning season. Support for this hypothesis is especially strong in smallmouth bass, where GL fish were 151 mm smaller than BR fish on average, suggesting a potential ontogenetic habitat shift of young GL fish into the lower river for feeding and/or refuge. Our study illuminates the complex dynamics shaping genetic structure in fragmented river systems and indicates that conclusions drawn for a single species cannot be generalized.

## 1 INTRODUCTION

Contemporary genetic structure of wild populations is the result of multiple evolutionary and ecological processes that often act simultaneously on overlapping but variable timescales. Some of these processes include allopatric divergence resulting from isolation in different glacial refugia (Bailey & Smith, 1981; Bernatchez & Wilson, 1998; Sepulveda‐Villet & Stepien, 2012), reproductive isolation resulting from spatial separation following recolonization via isolation by distance (Wright, 1943, 1946), population divergence as animals fill open habitats and adapt to different environmental conditions (i.e., isolation by adaptation/environment; Nosil et al., 2009; Orsini et al., 2013; Sexton et al., 2014), and genetic divergence resulting from fragmentation and recent barriers to gene flow (Keyghobadi, 2007). Disentangling the relative influence of these processes on contemporary genetic structure can be complex but is important for informing management and conservation action (Epps & Keyghobadi, 2015; Palsboll et al., 2007).

Dammed rivers are a well‐known example of fragmented systems with evolutionarily recent barriers to gene flow. Some studies have observed clear effects of human‐mediated fragmentation on the genetic structure of fish populations (Brauer & Beheregaray, 2020; Horreo et al., 2011; Raeymaekers et al., 2008; Yamamoto et al., 2004), while others have not (Clemento et al., 2008; Ruzich et al., 2019). For most systems, it can be difficult to disentangle the genetic effects of contemporary fragmentation from historic evolutionary processes (Epps & Keyghobadi, 2015; Ewers & Didham, 2006). One potential approach that can enhance our understanding of the relative impacts of natural vs. anthropogenic forces on genetic structure in fragmented rivers is to examine multiple species with varying life histories (e.g., Blanchet et al., 2010; Ruzich et al., 2019).

**FIGURE 1:**
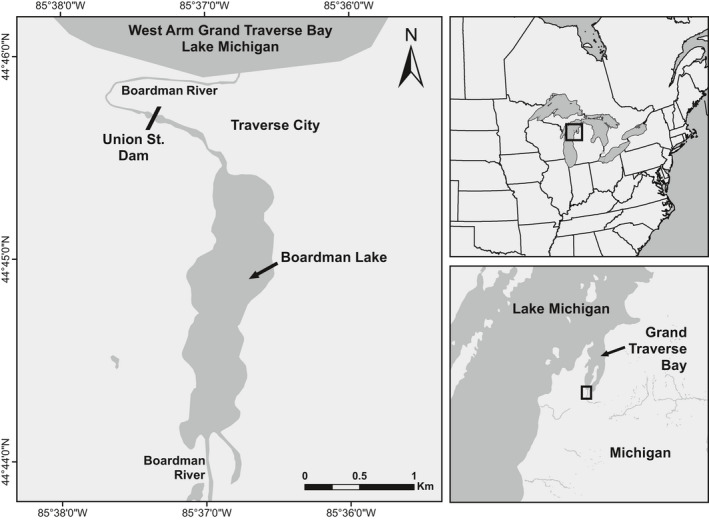
Sampling area in the lower Boardman River in Traverse City, MI, USA, which drains into northeast Lake Michigan. All “Upstream” sampled fish were collected in Boardman Lake, above the Union Street Dam. “Downstream” sampled fish were all captured in the Boardman River below the Union Street Dam, except for yellow perch which were collected from the West Arm of Grand Traverse Bay <1 km from the mouth of the river

Here, we investigate the relative influence of habitat fragmentation caused by a dam on the genetic structure of five fish species in the lower Boardman River, a tributary to the Laurentian Great Lakes. The Boardman River drains a 740 km^2^ watershed in the northwest of the Lower Peninsula of Michigan, USA (Figure 1). In its lower reach, the river flows through Boardman Lake, a natural drowned river mouth lake, before emptying into Grand Traverse Bay, an inlet of northeastern Lake Michigan. Most of the Boardman River watershed is isolated from Lake Michigan by the Union Street Dam, an earthen dam constructed in 1867 and located just 1.5 km upstream from the river mouth (Kalish et al., 2018). The dam has effectively blocked all upstream movement of most fishes with the exception of introduced Pacific salmonids which could ascend a pool and weir fishway that was operational between 1986 and 2019 (Daniel Zielinski, GLFC, personal communication).

The Boardman River drainage is part of the Laurentian Great Lakes, a system characterized by large‐scale habitat changes resulting from natural processes over the last several thousand years (Larson & Schaetzl, 2001). The Great Lakes were recolonized from multiple glacial refugia approximately 13,000 years ago after the Wisconsin glaciation, and broadscale genetic differences in fish populations exist among these glacial lineages (Borden et al., 2009; Sepulveda‐Villet & Stepien, 2012; Stepien et al., 2009). Since recolonization, the Great Lakes have been highly dynamic (e.g., experiencing drastic water level fluctuations; Johnston et al., 2004), and fish have colonized diverse habitats. Intraspecific variation in life‐history strategies including fluvial vs. adfluvial residence (Blumstein et al., 2018; Borden, 2008; Stepien et al., 2007) and spawning philopatry (Leung & Magnan, 2011; Stepien et al., 2009; Strange & Stepien, 2007; Wilson et al., 2016) has also created sympatric divergence and fine‐scale genetic structure among adjacent or overlapping populations in the Laurentian Great Lakes. For example, Chorak et al. (2019) observed that yellow perch occupying drowned river mouths (DRMs) connected to Lake Michigan were genetically distinct from Lake Michigan perch, even though the Lake Michigan perch utilized DRM habitats during part of the year. Since the arrival of European settlers, humans have drastically altered the Great Lakes ecosystem through various activities (Regier et al., 1999; Ricciardi & MacIsaac, 2000), such as the construction of thousands of dams (Januchowski‐Hartley et al., 2013). Contemporary genetic structure within Great Lakes fish species may thus be an artifact of multiple natural and anthropogenic processes that have occurred over both contemporary and evolutionary timescales.

We assessed the population genetic structure of five fish species that are native to the Laurentian Great Lakes and found both above and below the Union Street Dam: white sucker *Catostomus commersonii*, yellow perch *Perca flavescens*, walleye *Sander vitreus*, smallmouth bass *Micropterus dolomieu*, and rock bass *Ambloplites rupestris*. These species demonstrate varying life histories, generation times, and migratory behaviors (Becker, 1983; Scott & Crossman, 1985; Table 1). For example, walleye and white sucker generally exhibit distinct migrations up tributaries in the spring to spawn (Becker, 1983) and tend to demonstrate natal homing (Crowe, 1962; Werner, 1979). Smallmouth bass, rock bass, and yellow perch may also migrate and exhibit spawning site philopatry, but movements and degree of homing tend to fluctuate among populations and systems (Brown et al., 2009; Gerber & Haynes, 1988; Glover et al., 2008; MacLean & Teleki, 1977). Substantial life‐history variations also exist within our study species. For example, smallmouth bass and rock bass are known to demonstrate both lake and river life histories (Barthel et al., 2008; Gerber & Haynes, 1988; Noltie & Keenleyside, 1987), and these two ecotypes could remain genetically distinct even in the absence of geographic barriers (Borden, 2008; Euclide et al., 2020; Stepien et al., 2007). It is important to note that walleyes have been stocked extensively throughout the region including in Grand Traverse Bay and Boardman Lake (GLFC, 2020; MIDNR, 2020), and any natural genetic structure in the Boardman system may be masked by decades of stocking from non‐native sources. To our knowledge, no evidence exists that any of our four other study species have been stocked in the Boardman system or in Grand Traverse Bay.

**TABLE 1:** Summary of general life‐history traits of our study species in the upper Great Lakes region. Life‐history traits were derived from Becker (1983), Scott and Crossman (1985), and input from regional biologists (Daniel Zielinski GLFC, Dan Isermann USGS, personal communication)

Species	Reproductive strategy	Migration distance	Spawning philopatry	Life span (years)	Age at maturity (years)	Fecundity
Rock bass	Nest	Short	Med	6–10	2–3	Low–Intermediate
White sucker	Broadcast	Med‐Long	Med‐High	~20	2–8	Intermediate–High
Smallmouth bass	Nest	Short‐Med	Med‐High	~18	2–4	Intermediate
Yellow perch	Broadcast	Med	Low‐Med	8–12	2–4	Intermediate
Walleye	Broadcast	Med‐Long	Med‐High	5–20	2–6	High

Our overall goal was to determine whether any observed population structure was the result of historic evolutionary processes and/or fragmentation by the Union Street Dam. Specifically, we (1) explored the population structure and diversity of our study species above and below the dam using genotypes from thousands of loci generated with restriction site‐associated DNA (RAD) sequencing, (2) conducted genetic migration simulations to compare our empirical data to simulated data generated under a variety of divergence scenarios, and (3) evaluated the relationship between observed patterns in population genetic structure and ecological attributes such as fish size and date of capture. Our study illustrates the importance of including multiple species when investigating the relative influence of different factors on contemporary genetic structure. Additionally, our approach is highly applicable to other species and systems and should provide valuable information that will aid management of genetic diversity in fragmented habitats.

## 2 METHODS

### 2.1 Sample collection

We collected tissue samples from fish above and below the Union Street Dam for each of our five study species from 2017 to 2019. All sampling was performed with IACUC approval under University of Wisconsin—Stevens Point protocol number 2019.03.05. Fish below the dam were captured by boat electrofishing from the mouth of the Boardman River up to the Union Street Dam. Samples were taken during routine monitoring surveys, with the vast majority of fish being captured between April and July, and sampling spread relatively equally across 2017 and 2018, with only a few samples collected in 2019 (Figure S1). Field crews were unable to capture yellow perch in this river section, and we therefore acquired tissue samples from fish captured by ice anglers nearby (~1 km away) in Grand Traverse Bay during February 2018. Above the dam, fish were collected in Boardman Lake using experimental gillnets, mini fyke nets, or boat electrofishing. All above‐dam samples were collected in June 2019 with the exception of 14 walleyes collected in March 2018. Fish were identified to species, measured (mm total length), and tissue samples from the caudal or pelvic fins were collected and preserved in 95% ethanol. DNA was extracted from fin tissue with DNeasy^®^ 96 Blood & Tissue Kits (Qiagen).

### 2.2 RAD sequencing and SNP discovery

We prepared restriction site‐associated DNA (RAD) libraries with the *SbfI* restriction enzyme following the BestRAD procedure (Ali et al., 2016) and methods outlined in Ackiss et al. (2020) with minor modification. Prepared libraries were sent to Novogene for sequencing on the Illumina NovaseqS4 platform (PE150 chemistry). The resulting sequences were processed through the STACKS v2.3 software pipeline (Catchen et al., 2011, 2013) to demultiplex and filter raw reads, identify SNPs, and conduct genotyping. STACKS parameters for all species were as follows: *process_radtags* (‐e SbfI, ‐c, ‐q, ‐filter_illumina, ‐r, ‐‐bestrad, ‐t 140), *ustacks* (‐‐disable‐gapped, ‐‐model_type bounded, ‐‐bound_high 0.05, ‐M 3, ‐max_locus_stacks 4, ‐m 3, ‐H, ‐p 32), *cstacks* (‐n 3, ‐p 6 ‐‐disable_gapped). These STACKs parameters were derived based on review papers by Mastretta‐Yanes et al. (2015) and Paris et al. (2017) and have been shown to work well for similar fish species with nonduplicated genomes (e.g., Bootsma et al., 2020). SNPs genotyped in >30% of individuals (parameter flag: ‐r 0.3) were exported with the subprogram populations in variant call format (vcf) files. Filtering was then performed with vcftools v0.1.15 (Danecek et al., 2011) and included (1) removing loci genotyped in <70% of individuals, (2) removing individuals genotyped at <70% of loci, and (3) removing loci with a minor allele count less than 3. We then used the program HDPlot (McKinney et al., 2017) to investigate read ratio deviation between alleles as well as locus specific heterozygosity and removed loci with heterozygosity greater than 0.60 or a read ratio deviation greater than 5 and less than −5. HDPlot is helpful for identifying potentially duplicated loci as well as identifying any potential laboratory or bioinformatic errors (e.g., contamination, inappropriate STACKs parameters) that may influence data quality. Finally, only the SNP with the highest minor allele frequency on each tag was included in the final dataset because loci on the same RAD tag may be linked.

### 2.3 Genetic differentiation and diversity

To assess and visualize genetic differentiation within each species, we first conducted principal component analysis (PCA) using the R package adegenet (Jombart, 2008). We also estimated the number of ancestral populations (*K*) contributing to contemporary structure for each species using the program ADMIXTURE v1.3 (Alexander et al., 2009). We tested *K* from 1 to 5 with ADMIXTURE’s cross‐validation procedure and then plotted *K *= 2 through *K *= 5 for each species in R (Figure S2). We then calculated pairwise *F*_ST_ and summary statistics for each population using the R package diveRsity (Keenan et al., 2013). Summary statistics included allelic richness, observed heterozygosity (*H*_o_), expected heterozygosity (*H*_e_), and inbreeding coefficients (*F*_IS_). We tested the significance of each population comparison with a test for genetic differentiation (Goudet et al., 1996) conducted in GENEPOP (α = 0.01). Using the R package related (Pew et al., 2015; Wang, 2011), we tested the relatedness of individuals within each species using the Wang pairwise relatedness estimator (Wang, 2002). Any pair of individuals with a relatedness value greater than 0.4 was considered highly related (likely siblings or parent/offspring).

Effective population size (*N*_e_) of each population was estimated with the bias‐corrected linkage disequilibrium method (Hill, 1981; Waples, 2006; Waples & Do, 2010) in the software package NeEstimator v2.1 (Do et al., 2014) with a p‐crit of 0.05 (Waples et al., 2016). Physical linkage can bias estimates of *N*_e_ downward; therefore, we used genome resources to restrict comparisons to markers on different chromosomes when a genome assembly was available (yellow perch, walleye) and implemented a formula for correcting bias based on chromosome number for the other species (smallmouth bass, rock bass, white sucker; equation 1a in Waples et al., 2016). Alignments to the yellow perch genome for yellow perch and walleye, which shares the same karyotype (Danzmann, 1979), were conducted in BLASTN (Camacho et al., 2009); the best alignment for each locus was retained, and all alignments had e‐values <1 e^−51^. Chromosome numbers for species where genomes were not available are as follows: *N* = 23 for smallmouth bass (Beçak et al., 1971), *N* = 24 for rock bass (Avise & Gold, 1977), and *N* = 50 for white sucker (Beçak et al., 1973). *N*_e_ calculations using the linkage disequilibrium method can be biased slightly downward when individuals from multiple cohorts are included in the sample due to a slight Wahlund effect (7% downward bias on average; Waples et al., 2014), but this small bias should not greatly affect the interpretation of the *N*_e_ results.

**FIGURE 2:**
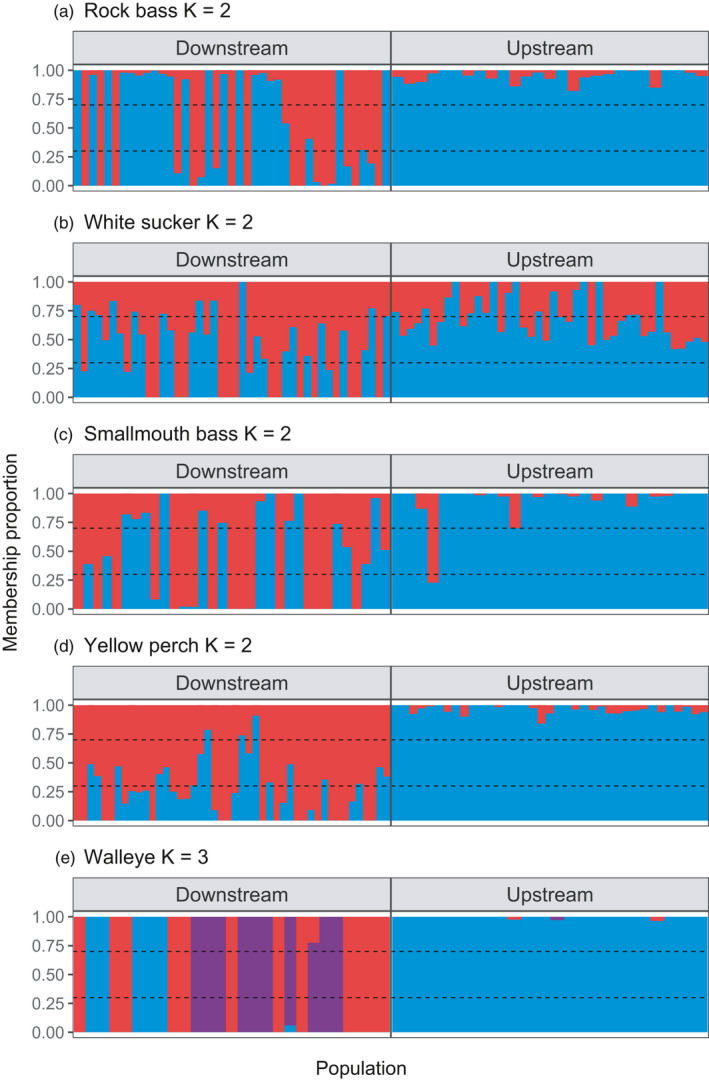
Genetic ancestry of all five study species captured either upstream or downstream of the Union Street Dam estimated with ADMIXTURE. Each vertical bar represents an individual, and color corresponds to ancestry proportions. All species (a–d) were best represented by two lineages (*K *= 2), except for walleye (e) which was best represented by three lineages (*K *= 3). Blue portions of ancestry correspond to the putative “Boardman River” (BR) genetic group, and red portions correspond to the putative “Great Lakes” (GL) genetic group. Walleye additionally split into a second “Great Lakes” group (GL2) which corresponds to the purple portion of ancestry. Horizontal lines at 0.3 and 0.7 represent cutoffs for determining whether an individual was potentially admixed (see Table 3)

For some species, visualization of PCA and ADMIXTURE plots suggested population groupings that were not consistent with our originally sampled “above dam” and “below dam” populations, but more broadly with what we hereafter refer to as a putative “Great Lakes” (GL) population and a putative “Boardman River” (BR) population (see Results for further explanation). To assess this unexpected structure and compare population‐level statistics using sampled populations and inferred populations, we reformed genetic groups based on population assignment from ADMIXTURE (Figure 2) to either the GL or BR population for each species. For rock bass, yellow perch, smallmouth bass, and white sucker, we assigned individuals to either the GL group or the BR group by using a membership proportion (Q‐score) cutoff of 0.5 from ADMIXTURE population assignment. For walleye, the PCA exhibited three distinct genetic groups (one BR population and two GL populations), and we assigned individuals to one of these three groups (BR, GL1, GL2) also using a membership proportion (Q‐score) cutoff of 0.5 from ADMIXTURE population assignment. We then recalculated summary statistics, *F*_ST_, and *N*_e_, and developed PCAs using these new genetic groups. Additionally, we estimated the proportion of putatively admixed individuals in each species by classifying individuals with a maximum Q‐score of less than 0.7 as putatively admixed (c.f. Ackiss et al., 2020). We recognize that the cutoff‐based method we used for population assignment may not capture the full extent of the structure in the dataset and that the existence of admixed individuals means that some will be assigned to a population even though they may have a Q‐score of only slightly above 0.5. Despite these limitations, we feel that our approach is appropriate and conservative given the complicated nature of our data and the relatively limited sample sizes. For example, we believe our approach is preferable to classifying putative admixed individuals as their own groups as this would serve to inflate the estimates of differentiation between putative BR and GL groups, and sample sizes for putative admixed groups would be very low.

We used the program BAYESCAN (Foll & Gaggiotti, 2008) to identify outlier loci potentially displaying signals of directional selection in each species except walleye, which were likely stocked from multiple nonlocal sources (see Results). BAYESCAN was run using the default parameters and a conservative false discovery rate of 0.01 to identify putative outliers. We then assessed the putative function of outliers by querying their sequences in the NCBI nucleotide and protein databases using BLAST. Only alignments with e‐values <10^−10^ were retained.

### 2.4 Comparison of empirical data to simulated migration scenarios

We simulated multiple demographic scenarios to investigate whether the levels of genetic differentiation that we observed between above‐ and below‐dam populations were consistent with recent isolation (i.e., isolation by the Union Street Dam) or longer‐term isolation that existed before construction of the dam. We did not consider walleye in this analysis as they were likely stocked from multiple nonlocal sources (see Results). To approximate the construction of a dam, we simulated scenarios where a barrier was placed between two panmictic populations 30 generations ago. We chose a generation value of 30 as our species have a generation time of approximately five years (Becker, 1983; Scott & Crossman, 1985), and the Union Street Dam was built approximately 150 years ago. We recognize that age at maturity and generation time does vary among species and that white sucker can mature later than our other study species (reaching maturity at age 2–8 compared to 2–4 for our other species; Table 1), but we chose to keep the generation time for all species consistent at five years for our simulations to avoid unnecessary complication to the interpretation of our data. It is important to note, therefore, that for white sucker the structure potentially caused by the dam may be slightly less than what our simulations indicate because their true generation time may be slightly higher than five years.

Once base simulation conditions were set, we explored the influence of different migration rates and *N*_e_s on genetic differentiation (*F*_ST_). Scenarios were simulated 10 times each in the coalescent‐based simulation in program fastsimcoal2 (Excoffier & Foll, 2011), and all simulations were initialized with two populations that initially exchanged a high number of migrants (m = 0.1 or a migration rate of 10%) before the barrier was constructed. Simulations approximated data from 15,000 unlinked loci with a maximum of two alleles and a mutation rate of 0.0001, and genetic statistics were assessed by sampling 50 individuals from each population. These parameters produced simulated datasets that were similar to the number of polymorphic loci (~5000 loci polymorphic in each simulation) and levels of heterozygosity (~0.2) in our empirical datasets. While this mutation rate is higher than expected for SNPs, guidelines for use of fastsimcoal2 indicate that matching empirical data is more important than achieving a realistic mutation rate, especially in situations like ours with short simulation timeframes where mutation rate will not be a major factor influencing genetic variation.

We simulated data using two values of *N*_e_ (100, 1000) that approximated the resulting *N*_e_ for the species we analyzed (*N*_e_ = 100 was similar to rock bass and smallmouth bass and *N*_e_ = 1000 was similar to yellow perch and white sucker). For each *N*_e_, we simulated a migration rate of zero and asymmetric migration rates of zero upstream migration and downstream migration rates of 0.01, 0.05, and 0.1, or 1%, 5%, and 10%. Although little information on downstream movement of cool water fishes, such as our study species, through dams is available, a recent study suggested that emigration of walleye out of Iowa reservoirs ranged between 2 and 26% (Weber & Flammang, 2019). Our values of downstream migration values bracket a similar range but do not exceed 10% because values higher than this result in highly genetically similar populations over short timescales. After conducting simulations, we used the R package diveRsity to estimate *F*_ST_ between simulated above‐ and below‐dam populations and visualized the results with boxplots.

### 2.5 Relationships between genetic structure and ecological data

To assess any correlation between genetic structure and ecological attributes, we first compared total lengths of fish between populations within each species. We made comparisons between the originally sampled above‐ and below‐dam populations and between the putative BR and GL genetic groups for each species. We evaluated test assumptions with the Shapiro–Wilk test for normality, visualization of Q‐Q plots, and Levene's test for equality of variances. Length comparisons were performed using Student's two‐sample *t* tests in instances where variances were equal, and by using Welch's unequal variances *t* test when variances were unequal. For length comparisons among walleye genetic groups (three populations rather than two), we performed a one‐way ANOVA and then post hoc Tukey's HSD test for multiple comparisons. Because we saw a mix of genetic groups within each sampling location for smallmouth bass, rock bass, and white sucker, we further split fish into groups for each combination of genetic group and sample location and compared lengths for each species using a one‐way ANOVA and post hoc Tukey's HSD after testing assumptions using the same methods listed above. For all tests, we used α = 0.05.

We also qualitatively assessed whether the proportion of GL and BR fish within each sampling event varied across sample date because several collection events occurred for most species. We plotted the proportion of each genetic group within each sampling event for each species and visually assessed how proportions differed across dates. It is important to note that sampling was not performed evenly across time, sampling effort may not have been consistent between events, and some dates have very low sample sizes (as few as one fish in some instances); therefore, we did not perform a statistical analysis for this assessment.

## 3 RESULTS

### 3.1 Quality control

A total of 428 individuals were RAD sequenced and 346 remained after filtering (Table S1). We successfully genotyped 49 to 86 individuals per species, with the smallest sample size per population in walleye, where we genotyped 22 individuals sampled above the dam and 27 individuals below (see Table 2; Table S1 for information on number of individuals sampled and genotyped). The average number of reads per individual was generally high and ranged from 7,768,406 in yellow perch to 14,511,218 in white sucker. The percent of individuals genotyped per species ranged from 62% (walleye) to 89.6% (white sucker), with all species except walleye displaying a retention rate >85%. The low genotyping rate in walleye was likely a function of low sample quality in the above‐dam samples. After filtering, missingness in individuals averaged 12.5% across all species and ranged from 9.3% in walleye to 16.3% in white sucker (Table S1). The number of putative SNPs identified in each species ranged from 3812 in yellow perch to 38,126 in white sucker with an average about 13,000 SNPs per species (Table 2).

**TABLE 2:** Summary of RADSeq data, population information, and genetic diversity metrics calculated for our five study species in the lower Boardman River. Summary statistics were calculated both for original sample populations (below dam and above dam) and for putative genetic groups assigned from ADMIXTURE (Great Lakes group and Boardman River group). *N* SNPs is the total number of putative SNPs detected within each species after filtering. *N* outlier loci are the number of highly differentiated loci within each species; related pairs are the number of pairs within a species with a Wang relatedness estimation of 0.4 or higher (siblings or parent/offspring). Sample size is the number of individuals successfully genotyped in a given group. *F*_ST_ is the measure of genetic differentiation, AR is allelic richness, *H*_O_ is observed heterozygosity, *H*_E_ is expected heterozygosity, *F*_IS_ is the inbreeding coefficient, and *N*_e_ is effective population size. Avg TL is the average total length of individuals within a group

Species	*N* SNPs	*N* outlier loci	Related pairs	Group	Sample size	*F*_ST_	AR	*H*_o_	*H*_e_	*F*_IS_	*N*_e_	*N*_e_ CI Low	*N*_e_ CI High	Avg TL (mm)	SD
Rock bass	8655	2	4	Original population											
				Below dam	41	0.0261	1.983	0.317	0.341	0.063	24.9	24.9	24.9	154	40.6
				Above dam	27		1.978	0.315	0.32	0.01	78.2	77.5	78.8	164	31.8
				Genetic group from ADMIXTURE											
				Great Lake	20	0.1021	1.935	0.316	0.32	0.01	196.9	191.9	202.2	135	36.6
				B. River	48		1.964	0.316	0.322	0.016	104.9	104.3	105.5	167	33.8
White sucker	38,126	11	0	Original population											
				Below dam	44	0.0046	1.987	0.258	0.298	0.12	1246.7	1230.2	1263.7	443	54.3
				Above dam	42		1.989	0.257	0.298	0.122	693.1	687.6	698.7	477	68.2
				Genetic group from ADMIXTURE											
				Great Lake	32	0.0092	1.965	0.255	0.255	0.118	831.3	819.7	843.2	426	54.3
				B. River	54		1.982	0.26	0.299	0.121	1117.9	1107.3	1128.8	480	60.2
Smallmouth bass	11,044	2	4	Original population											
				Below dam	33	0.063	1.981	0.291	0.308	0.049	14.1	14.1	14.2	199	95.8
				Above dam	27		1.967	0.301	0.307	0.019	66.4	66.0	66.8	404	87.4
				Genetic group from ADMIXTURE											
				Great Lake	20	0.1326	1.887	0.276	0.269	−0.021	75.3	74.5	76.1	192	112.5
				B. River	40		1.962	0.306	0.312	0.02	103.0	102.5	103.6	341	121.9
Yellow perch	3812	0	0	Original population											
				Below dam	46	0.0346	1.935	0.265	0.266	0.004	801.3	738.4	875.8	253	28.4
				Above dam	37		1.964	0.268	0.27	0.007	8444.9	4066.2	∞	209	60.3
				Genetic group from ADMIXTURE											
				Great Lake	41	0.0371	1.925	0.264	0.263	−0.003	1977.1	1599.2	2587.1	254	28.1
				B. River	42		1.972	0.269	0.272	0.011	8250.8	4269	118,434	213	58.5
Walleye	4470	NA	2	Original population											
				Below dam	27	0.0671	1.964	0.26	0.278	0.054	41.1	40.4	41.8	574	141.8
				Above dam	22		1.887	0.26	0.262	0.001	142.8	133.1	154	397	70.8
				Genetic group from ADMIXTURE											
				Great Lake 1	12	0.1053^a^	1.81	0.262	0.259	−0.02	1492.2	898.5	4370	495	171.7
				Great Lake 2	10		1.787	0.259	0.249	−0.04	26.0	25.6	26.4	662	68.6
				B. River	27		1.815	0.259	0.263	0.009	221.0	213.4	229.1	430	99.1

^a^ Pairwise *F*_ST_ values among walleye groups: GL1 − GL2 = 0.057, BR − GL1 = 0.112, BR − GL2 = 0.118.

### 3.2 Genetic differentiation and diversity

**FIGURE 3:**
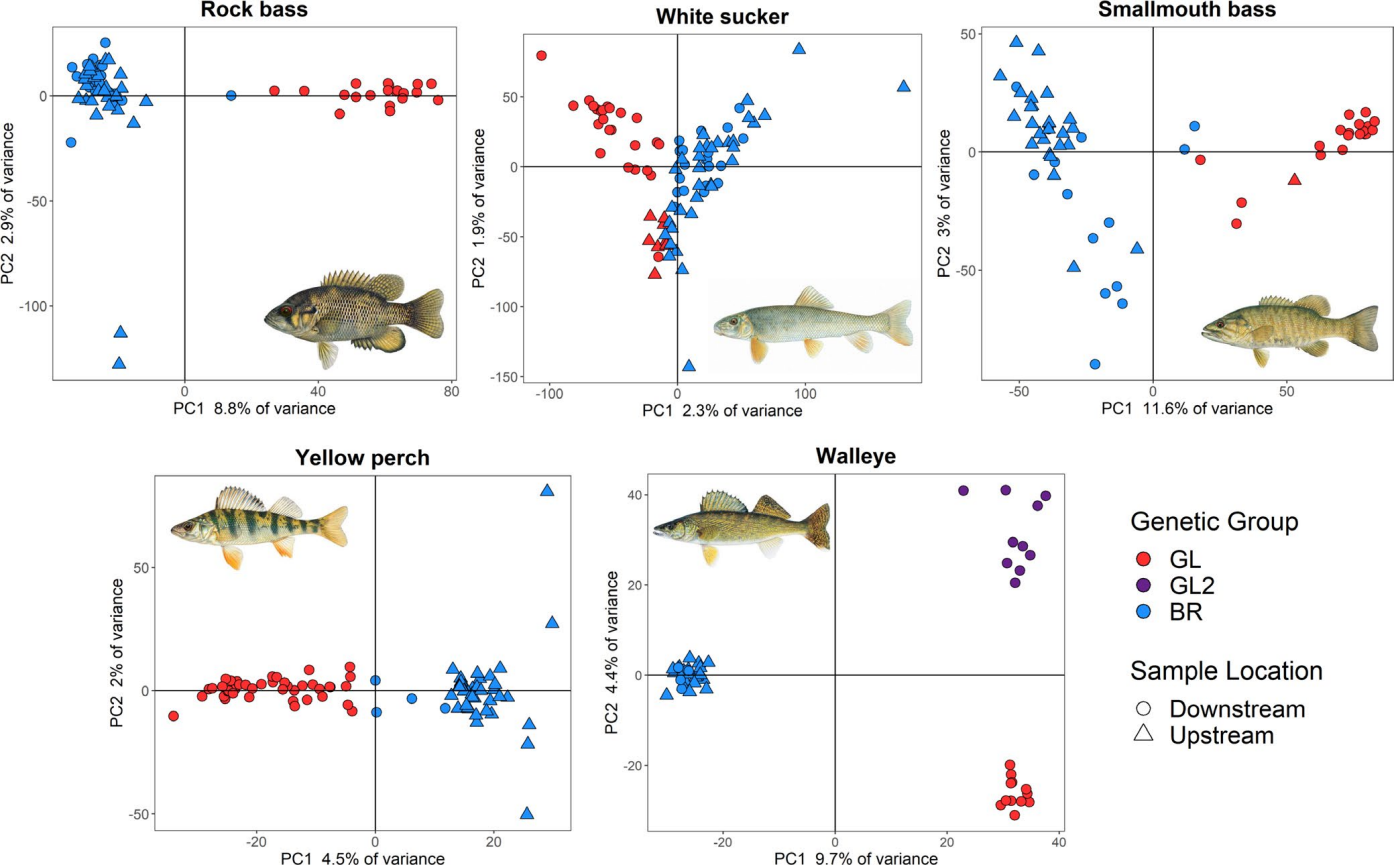
Principal component analysis (PCA) plots for all five study species (pictured in each plot). The percentage of variance explained by PC1 and PC2 is labeled on the x‐ and y‐axes, respectively. Colors correspond to putative genetic group determined by ADMIXTURE assignment, with blue corresponding to the Boardman River (BR) group and red corresponding to the Great Lakes (GL) group. Walleye split into a third genetic group (GL2 in purple), which appears to be associated with the Great Lakes but is distinct from the first GL group. Circles correspond to individuals captured downstream of Union Street Dam, and triangles correspond to individuals caught above the dam. Fish illustrations were created by Joseph Tomelleri and used with permission

Visualization of ADMIXTURE plots (Figure 2; Figure S2) and PCAs (Figure 3) suggested unexpected genetic groupings not consistent with original population designations (above‐ and below‐dam) for most species. However, the degree of correspondence between sampled population and genetically inferred population differed substantially across species. The best supported number of ancestral populations (*K*) from ADMIXTURE was 2 for all species except white sucker (*K *= 1). However, white sucker did display subtle evidence of population structure at *K *= 2 and the same subtle structure was apparent in the PCA. Therefore, we split white sucker into two genetic groups based on population assignment from ADMIXTURE as we did with rock bass, yellow perch, and smallmouth bass. For walleye, PCA and ADMIXTURE demonstrated three distinct groupings, with two GL groups separated from the BR group along PC1 and separated from each other along PC2. Although *K *= 2 was the best fit according to ADMIXTURE cross‐validation for walleye, we suspect this was because the upstream group was highly diverged from both downstream groups due to stocking (see below), and we therefore retained the three genetic groups for plots and analyses.

We also observed highly variable levels of putative admixture across species, ranging from no putatively admixed individuals in walleye to over 50% putatively admixed individuals in the above‐dam samples of white sucker (Table 3). As expected, levels of putative admixture generally corresponded with observed genetic structure, with the most highly structured species (walleye, rock bass, smallmouth bass) displaying lower levels of admixture compared to species with lower genetic structure (yellow perch and especially white sucker). It is difficult to disentangle whether high admixture is the result of true mixing of genetically differentiated individuals or an inability to resolve subtle population structure and it is likely that both of these factors are interacting in our dataset.

**TABLE 3:** Estimates of putative admixed individuals for each species. Individuals were classified as putatively admixed if they had a maximum Q‐score <0.7 based on ADMIXTURE analysis (see Figure 3). Downstream samples were from fish captured below the Union Street Dam, and upstream samples were from fish captured above the dam

Species	Number putative admixed		% putative admixed	
	Downstream	Upstream	Downstream	Upstream
Rock bass	3	0	7%	0%
White sucker	14	24	32%	57%
Smallmouth bass	5	0	15%	0%
Yellow perch	14	0	30%	0%
Walleye	0	0	0%	0%

After reforming populations using ADMIXTURE assignments, several general patterns became apparent: (1) For rock bass and smallmouth bass, we generally found a single and relatively homogenous Boardman River (BR) group above the dam and mixture of the BR group with the putative Great Lakes (GL) group below the dam. Both of these species also displayed some evidence of putative admixture or representation from more than two populations. In particular, the smallmouth bass data displayed evidence for one or two other intermediate genetic groups (or possibly hybrids). These groups likely correspond to unsampled populations in Lake Michigan, but we did not analyze them separately due to low sample sizes. (2) For white sucker, we found a somewhat similar but much weaker pattern of genetic structure compared to rock bass and smallmouth bass. Specifically, we observed a group of ~20 individuals sampled below the dam that appeared to be differentiated from other individuals in the study and may represent a GL group, but we also observed high levels of admixture in the other samples and very low genetic structure overall. (3) For yellow perch, our above‐ and below‐dam sample sites generally coincided with observed population structure, with low but significant structure observed between samples taken in Traverse Bay and Boardman Lake. However, we did observe that 30% of individuals in the downstream collection were putatively admixed, indicating that either our data do not have the resolution to consistently differentiate Boardman Lake and Traverse Bay yellow perch or that migrants from Boardman Lake have interbred with the Traverse Bay population. (4) For walleye, we observed three distinct genetic groups, a BR group consisting of mostly upstream fish as well as five fish caught downstream that likely originated in Boardman Lake and passed downstream through the dam, and two distinct GL groups consisting solely of individuals caught below the dam. Investigation of the stocking history for this system suggests these three groups are likely the product of decades of stocking from multiple sources (GLFC, 2020; MIDNR, 2020). These records show Boardman Lake was stocked with walleyes from New York (now our BR group), and Grand Traverse Bay has been stocked repeatedly from two main sources, Muskegon and Little Bay de Noc (now our GL1 and GL2 groups), which are both Lake Michigan systems but are located approximately 280 km across the lake from each other and are thus likely to be genetically distinct. No genetically intermediate individuals were observed in our walleye populations, even between the two GL populations, suggesting that these groups do not interbreed or that successful reproduction is not occurring below the dam where populations are mixed.

Genetic differentiation (*F*_ST_) for all species was highly significant in both original population groupings (i.e., above vs. below the dam) and population groupings based on ADMIXTURE analysis. The *F*_ST_ of original populations ranged from 0.0046 (white sucker) to 0.0671 (walleye; Table 2) but generally increased when we reformed genetic groups. Rock bass had a large increase, from 0.0261 to 0.1021, as did smallmouth bass which changed from 0.063 to 0.1326. White sucker *F*_ST_ was the lowest of all species in both scenarios and went from 0.0046 to 0.0092. Yellow perch had the smallest change, from 0.0346 to 0.0371, likely because population assignment did not change as drastically as our other study species. Walleye *F*_ST_ changed from 0.0671 (pairwise) to 0.1053 (overall). Because we changed walleye from the original two populations to three genetic groups, we assessed pairwise *F*_ST_ between the three groups in addition to overall *F*_ST_. The two GL groups had a pairwise *F*_ST_ of 0.057, and the BR group had a pairwise *F*_ST_ of 0.112 and 0.118 between GL1 and GL2, respectively, providing further evidence to support our hypothesis that the BR group is derived from a highly divergent out‐of‐basin source (New York).

Observed heterozygosity (*H*_o_) differed by 0.01 or less between original above‐ and below‐dam populations for all species (Table 2). When we reassigned populations to the BR and GL genetic groups, heterozygosity again differed by 0.01 or less for all species except smallmouth bass, where *H*_o_ was 0.276 for the GL group and 0.306 for the BR group. Allelic richness demonstrated similar patterns, with little variation among groups outside of regrouped smallmouth bass populations, where allelic richness differed by ~0.05 between the GL and BR groups. Estimates of *F*_IS_ were near zero (between −0.04 and 0.02) for all populations grouped based on ADMIXTURE except for white sucker, where *F*_IS_ was ~0.12 in all populations for all groupings.

We conducted a number of analyses to attempt to understand why *F*_IS_ was elevated in white sucker and hypothesize that this trend was the result of a genome duplication in Catostomidae (Uyeno & Smith, 1972). Specifically, we first tested different ‐M (mismatches allowed) values in STACKs; values tested were 1, 3 (original value), and 7. The pattern of high *F*_IS_ was present at all ‐M values. We then conducted analyses outside of STACKs by aligning quality‐filtered reads for sequenced individuals to a small number of loci with high *F*_IS_ values and compared read counts and genotypes derived from STACKs with these alignments. We found that many loci had a large number of reads for a sequence that was a close or exact match to the target allele and a sequence that was similar but substantially different (potentially a paralog). Many of these loci were called as homozygotes in STACKs, leading to high frequencies of alternate homozygotes, few heterozygotes, and high *F*_IS_ values. Unfortunately, few genomic resources exist for Catostomidae, making it difficult to confirm our hypotheses, but we hope that sequenced genomes for this family will clarify the pattern in the future. We do, however, believe that analyses based on our full dataset are robust, as we analyzed population structure with three different datasets (loci with *F*_IS_ < 0.2, *F*_IS_ < 0, and *F*_IS_ > 0) and found the same patterns (*F*_ST_ values within 0.0006 of values in the overall datasets and extremely similar patterns of population divergence in PCAs). We therefore decided to retain our full dataset for all analyses with the caveat that true *F*_IS_ values may be lower than our estimates.

Estimates of *N*_e_ ranged from 14 to over 12,000 and generally increased when populations were grouped according to ADMIXTURE rather than sampling location (Table 2). Rock bass and smallmouth bass tended to have the lowest *N*_e_ estimates, between 75 and 192 when populations were grouped by ADMIXTURE. White sucker and yellow perch had much larger *N*_e_ estimates near or above 1000 for all population groupings, with estimates as high as 8845 for yellow perch samples taken above the dam. Estimates of *N*_e_ for walleye varied substantially between the three genetic groups defined by ADMIXTURE, with estimates of 221 for the BR group, 1492 for the GL1 group, and 26 for the GL2 group. It is important to note that two related pairs of individuals were found in the GL2 group (i.e., four individuals out of 10 were related, see below) and this may have potentially reduced the *N*_e_ estimate for this group. Estimates of *N*_e_ were generally similar between BR and GL groups, but slightly higher estimates were observed in the BR group for smallmouth bass, white sucker, yellow perch, and a slightly lower estimate was observed in the BR group for rock bass. Alignments to the yellow perch genome were successful for 1970 tags (44%) in walleye and 3746 tags (98%) in yellow perch; these alignments were used to remove bias due to physical linkage when calculating *N*_e_ for these species.

Related individuals (parent–offspring or full siblings) were present in some but not all species in our dataset, with yellow perch and white sucker containing zero related pairs, walleye containing two, and smallmouth bass and rock bass each containing four (Table S2). Related pairs were always captured in the same sampling area (i.e., above or below the dam) and belonged to the same genetic group. In walleye, both related pairs were sampled downstream and belonged to group GL2. In smallmouth bass, three of four pairs were sampled above the dam and belonged to the BR group, and the other pair was sampled below the dam and belonged to the GL group. Three of four rock bass pairs were sampled below the dam, one was sampled above the dam, but all pairs assigned to the BR group. Most related pairs appeared to be siblings as they were generally similar in length, but three of four pairs in smallmouth bass and one of four pairs in rock bass differed substantially in length and may have represented parent–offspring pairs. The number of related individuals in each species appeared to be somewhat related to *N*_e_, as species with larger *N*_e_s (white sucker, yellow perch) did not have any related individuals. We retained related pairs for all analyses as we have no reason to believe that rates of relatedness that we observed are nonrepresentative of each population (Waples & Anderson, 2017).

Outlier tests identified a relatively small number of highly differentiated loci: zero loci in yellow perch, two in smallmouth bass and rock bass, and 11 in white sucker (Table 2; Figure S3). In general, distributions of *F*_ST_ were relatively continuous and did not reveal large breaks with highly differentiated loci. Additionally, the fact that only two populations were included in each analysis likely led to low power for detecting outliers. We were able to successfully align four out of 15 outliers to protein sequences, one locus for smallmouth bass, one locus for rock bass, and two loci for white sucker (Table S3). Our most notable alignment was the locus in rock bass, which aligned to an immunoglobulin‐like protein that may be involved in immune system function. The other loci aligned to a transposable element, and genes coding for an integrase and elongation factor (Table S3).

### 3.3 Comparison of empirical data to simulated migration scenarios

**FIGURE 4:**
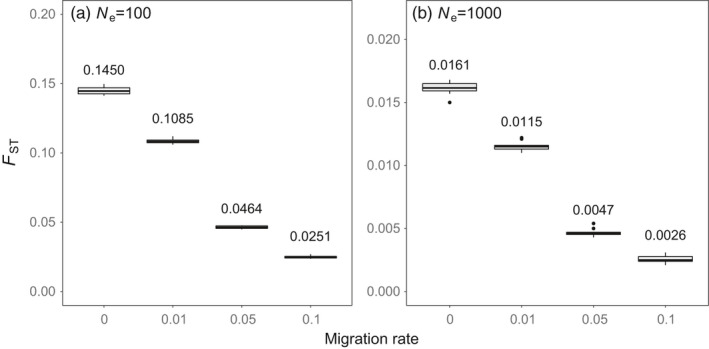
Results from simulated migration scenarios estimating genetic differentiation (*F*_ST_) across a barrier over the approximate life span of the Union Street Dam (30 generations with an estimated generation time of 5 years). We tested populations with *N*_e_s of 100 (a) and 1000 (b), and migration rates of 0, 0.01, 0.05, and 0.1, simulating each scenario 10 times and plotting resulting *F*_ST_ outputs. Mean *F*_ST_ values for each scenario are displayed over each boxplot. Migration was fully asymmetric (upstream to downstream) in all scenarios, with no upstream migration

Simulations of zero and asymmetric migration (m) revealed that after 30 generations, two populations with *N*_e_s of 100 should display *F*_ST_ values averaging 0.145, 0.109, 0.046, and 0.025 under migration rates of 0, 0.01, 0.05, and 0.1, respectively (Figure 4a). The two species with *N*_e_ close to 100, rock bass and smallmouth bass, displayed *F*_ST_ values of 0.13 and 0.10, respectively, which most closely match simulations with either no or very low (m = 0.01) migration. This indicates that if the population differentiation that we observed in these species was caused by the Union Street Dam, migration between above‐ and below‐dam populations would need to be extremely small (near 1%). However, we observed a large number of individuals of both GL and BR origin below the dam. We therefore hypothesize that the most probable explanation for the patterns of genetic structure that we observed in rock bass and smallmouth bass is the existence of separate BR and GL populations, with GL populations mixing with BR populations in the lower river but rarely interbreeding.

Simulations of two populations with *N*_e_s of 1000 unsurprisingly produced smaller *F*_ST_ values, averaging 0.016, 0.012, 0.005, 0.003 under migration rates of 0, 0.01, 0.05, and 0.1, respectively (Figure 4b). The two species with *N*_e_s near 1000, white sucker and yellow perch, displayed *F*_ST_ values of 0.009 and 0.037, respectively. It is plausible that genetic differentiation similar to the levels observed in white sucker could occur in the timeframe since the dam was built if migration was low (observed *F*_ST_ for white sucker similar to the value observed for m = 0.01). However, the fact that genetically similar white suckers are found above and below the dam suggests that some other factor may be causing the structure that we observed. Specifically, the ~20 fish sampled downstream that were identified by PCA and ADMIXTURE appear somewhat diverged from the rest (upper left of PCA Figure 3, Q‐scores near 0 in downstream portion of ADMIXTURE plot Figure 2b) and may represent a potentially unique genetic group. We hypothesize that these ~20 unique GL fish may spawn lower in the Boardman drainage while the remaining fish from above and below the dam represent a single population that potentially spawn higher in the watershed (or at least did so prior to dam construction). We also hypothesize that the admixture observed in white suckers is more a function of lack of resolution between genetic groups that are only subtly differentiated rather than signals of true admixture (i.e., interbreeding). For yellow perch, the observed *F*_ST_ value (0.0371) was over two times higher than the *F*_ST_ estimate for the m = 0 scenario, therefore providing the strongest evidence for differentiation before dam construction among our study species. However, we did observe some putative admixture among the two yellow perch groups below the dam, suggesting that some gene flow may be occurring, although based on our simulation results it does not appear to be enough to homogenize the populations.

### 3.4 Relationships between genetic structure and ecological data

**FIGURE 5:**
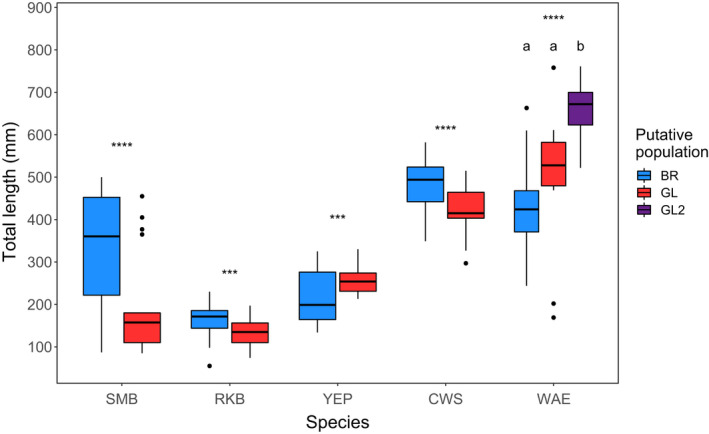
Differences in total length in mm between putative genetic groups within each study species in the lower Boardman River. All species were assessed using *t* tests except for walleye, where we used ANOVA and Tukey's HSD as this species contained three genetic groups. All comparisons were significantly different at α = 0.05. *p ≤ 0.05, **p ≤ 0.01, ***p ≤ 0.001, ****p ≤ 0.0001

Lengths of fish were significantly different between BR and GL groups defined by ADMIXTURE for all species (Figure 5). Walleye in the GL2 group were significantly longer than the other two groups but there was no significant difference between the lengths of GL1 and BR groups. For yellow perch, the GL group was longer than the BR group, but for the other three species (smallmouth bass, rock bass, and white sucker), fish from the GL group were shorter than those from the BR group. The most striking differences in lengths were in smallmouth bass, where fish from the GL group were 151 mm shorter on average compared to the BR group (Figure 5; Table 2). Differences were smaller for other species ranging from ~30 mm in rock bass to ~55 mm in white sucker. Two of the three walleye groups did have large differences in lengths, with GL2 averaging 662 mm and BR averaging 430 mm (a difference of ~230 mm), but we suspect this was because fish in GL2 were primarily large spawning fish that were sampled in the river below the dam.

For length comparisons among combinations of genetic group and sample location, the GL‐downstream white suckers were significantly shorter than both the BR‐downstream and the BR‐upstream groups (Figure S4). The same pattern held true for rock bass. As above, the most striking differences in lengths were observed in smallmouth bass. Both the BR and GL fish caught below the dam were significantly shorter on average (222 mm and 182 mm, respectively) compared to the BR fish caught upstream (405 mm). Within both genetic groups caught below the dam, however, there were a small number of larger fish that were of spawning size (350–455 mm; Figure S4). We did not make comparisons with the GL‐upstream group of smallmouth bass as it contained a sample size of 1.

For most species, the main general pattern that emerged when we visually assessed the proportion of each genetic group by sampling date was the relatively higher numbers of fish caught below the dam during months when spawning was likely occurring (Figure S1). For example, 36 white suckers were caught below the dam in April 2019, and only five were caught in June 2019. For most species, however, the number of fish sampled on each date was either low (walleye), clustered into one major sampling effort per population (yellow perch), or proportions of fish in each genetic group were similar (rock bass and white sucker). Therefore, we did not observe potentially meaningful patterns in general. For smallmouth bass, however, it appeared that GL fish made up a greater proportion of the fish sampled below the dam during May 2017 compared to July 2017 or September 2018, which each had a comparatively higher proportion of BR fish, but lower numbers of fish overall (Figure S1).

## 4 DISCUSSION

While dams are ubiquitous worldwide, few studies have assessed the genetic structure of multiple fish species across a small spatial scale (<5 km) in a dammed river. Our results demonstrate some genetic differentiation on the scale of a few km within all five of our study species (rock bass, white sucker, smallmouth bass, yellow perch, and walleye) in the fragmented lower section of the Boardman River. Patterns of differentiation were variable across species and are likely the result of multiple natural and anthropogenic processes that include fragmentation by the Union Street Dam but were likely more influenced by other factors. In general, we observed three major patterns: (1) in rock bass, smallmouth bass, and, to a much lesser extent, white sucker, we observed that individuals sampled above the dam were generally genetically homogenous and individuals below the dam seem to represent a mixed stock of fish from the above‐dam (i.e., Boardman River or BR) and below‐dam (i.e., Great Lakes or GL) genetic groups, with some admixture among groups, especially in white sucker. (2) In yellow perch, we observed significant but subtle population structure between fish sampled above the dam and in Traverse Bay with a large degree of putative admixture between groups, which may be the result of either true admixture, a lack of resolution in the data, or a combination of both factors. (3) In walleye, we observed high genetic differentiation caused by historic stocking from multiple geographical locations, and no apparent reproduction between the different genetic groups. Our overall results also suggest that, for at least some of our species, individuals from the putative GL genetic group leave their natal habitat in Lake Michigan and enter the lower Boardman River in certain life stages where they coexist with individuals from the putative BR genetic group. Before the Union Street Dam was constructed, both putative BR and GL fish within our study species were able to move freely throughout the Boardman River watershed and Grand Traverse Bay, and each genetic group likely used these different habitats during specific times of the year or during different stages of growth and development. Without our genetic assessment, the overlap of distinct genetic groups in some of our study species such as rock bass and smallmouth bass in the lower Boardman River would have likely continued undetected. These results also highlight the importance of tributary and river mouth habitats for preserving fish populations in the Great Lakes and emphasize that a conservation approach centered around maintaining high connectivity and habitat quality of tributaries is vital for ecosystem health. Finally, our results highlight the fact that patterns of genetic structure can be complex and highly variable across species and caution against over‐interpretation based on data from one or even a few species.

### 4.1 Hypotheses for potential drivers of observed population structure

While geographic isolation is often the largest factor influencing the landscape of genetic differentiation, research has highlighted that isolation by distance (IBD) may not always be the main variable driving contemporary population genetic structure. For example, adjacent populations may experience isolation by adaptation (IBA), in which gene flow is reduced as a result of local adaptation along differing ecological or environmental gradients (Nosil et al., 2009; Orsini et al., 2013; Rasanen & Hendry, 2008). IBA can occur across small spatial scales in the absence of geographic barriers to dispersal, revealing cryptic and often unexpected genetic structure that does not necessarily follow an IBD pattern. For example, Nosil et al. (2008) discovered that ecotypes of walking stick insects (*Timema cristinae*) adapted to different host species exhibit IBA at both neutral loci and loci under putative selection. IBA has also been documented in aquatic systems, including a study by Bond et al. (2014), who detected strong differentiation between freshwater and estuarine Dolly Varden (*Salvelinus malma*) populations within a small watershed lacking physical barriers, and hypothesized that IBA due to varying selective pressures across different environments was the main driver of observed structure.

We hypothesize that genetic structure in two of our study species displaying high differentiation, rock bass and smallmouth bass, may have been at least partially driven by IBA after deglaciation in the region, long before construction of the Union Street Dam. Great Lake and tributary habitats differ in a number of attributes including temperature, nutrient levels, and composition of prey species (Bhagat & Ruetz, 2011; Brazner & Beals, 1997; Höök et al., 2007; Janetski & Ruetz, 2015), possibly leading to differentially adapted populations of fish across relatively small spatial scales. Other recent studies throughout the Great Lakes have also revealed fine‐scale genetic structure between fish populations inhabiting main lakes and tributaries, suggesting that historical divergence through IBA may be more common within the Great Lakes and their tributaries than previously thought. For example, Euclide et al. (2020) detected strong genetic structure in Lake Michigan smallmouth bass across small spatial scales (10–30 km), which often correlated to differences in habitat type rather than geographic distance, and found that gene flow between lake and river sites was low, even though individuals from the two habitat types likely mixed outside of the spawning season. Additionally, Chorak et al. (2019) observed genetic structure in yellow perch populations between Lake Michigan and connected DRM habitats, even though the different populations overlapped during parts of the year. Taken together, our findings and those of past studies suggest that, for some Great Lakes species, distinct tributary and Great Lakes populations may have adapted to different habitat types and historically maintained low levels of gene flow even before the construction of anthropogenic barriers.

Other mechanisms, such as natal homing and site fidelity, can help to create, increase, and maintain differentiation (Lin et al., 2008), and we suspect this may be occurring in our study system as well. Both rock bass and smallmouth bass tend to occupy relatively small home ranges and exhibit spawning site fidelity (Gerber & Haynes, 1988; MacLean & Teleki, 1977; Ridgway et al., 1991), which could increase or maintain differentiation. White suckers exhibit distinct spawning migrations into tributaries in the spring to spawn and also display natal homing (Doherty et al., 2010; Geen et al., 1966; Werner, 1979). Before the construction of the Union Street Dam, the resident BR population of white sucker likely spawned in tributaries upstream in the watershed, while the GL fish may have spawned lower in the drainage, thus reducing gene flow with the BR group. This hypothesis is consistent with the differentiation we observed between the white sucker genetic groups. Yellow perch are primarily a lentic species and generally do not exhibit spawning migrations intro tributaries like our other species but do exhibit broad natal homing within their resident system, which can lead to genetic differentiation between spawning groups (Leung & Magnan, 2011; Parker et al., 2009; Sepulveda‐Villet et al., 2011). Our results indicate that subtle genetic structure does exist between BR and GL groups of yellow perch, and this structure could have been caused by natal homing. However, the structure we observed in this species is relatively weak compared to some of our other species (*F*_ST_ = 0.037), and we also observed substantial admixture in samples taken from Traverse Bay. This admixture may be the result of gene flow from Boardman Lake, a lack of resolution due to the large *N*_e_s and correspondingly low structure in yellow perch, or a combination of these two factors.

Although fragmentation by artificial barriers can impact the genetic structure and diversity of fish populations (Brauer & Beheregaray, 2020), especially in migratory species with strong natal homing like salmonids (Horreo et al., 2011; Samarasin et al., 2017; Wofford et al., 2005; Yamamoto et al., 2004), our data do not suggest that the Union Street Dam is the primary driver of genetic structure in the Boardman system. The construction of the dam happened relatively recently in evolutionary terms (~150 years ago). To observe any substantial genetic impact over the lifetime of this dam, affected fish populations would need to be relatively small and there would have to be very little downstream gene flow through the dam (as seen in our simulation results and also suggested by Hoffman et al., 2017; Keyghobadi, 2007; Selkoe et al., 2015). For most of our study species, our simulations demonstrated that if each species consisted of one genetically homogenous population before dam construction, a migration rate of 0% or 1% would have been necessary to produce the *F*_ST_s that we observed. However, based on the high mixing of the two populations below the dam for most species, and the likelihood that significant downstream movement of juveniles through the dam occurs (as seen in the BR walleye caught below the dam and in the admixed below‐dam individuals of other species), a nonexistent or extremely low level of downstream gene flow seems highly unlikely in this system, and therefore, it is improbable that the dam alone caused the differentiation we observed. However, the Union Street Dam is definitely a barrier to upstream migration and has substantially altered connectivity in this system. Thus, while we believe that most of the genetic structure that we observed in our study species formed before the dam was built, the dam has likely influenced genetic structure since it was built and will continue to influence structure until it is removed and/or fish passage is facilitated.

We derived multiple hypotheses based on the complex patterns of genetic structure across species in our dataset, all of which could be tested further with additional sampling. First, sampling spawning populations in the Great Lakes and comparing them to samples from the Boardman River drainage would confirm whether the genetic groups that we putatively assigned as Great Lakes origin do indeed represent Great Lakes spawning populations. Second, sampling additional loci to conduct a more complete genome scan could shed light on whether peaks of adaptation exist in our species. Although we did find some limited evidence of adaptation in outlier tests, our RAD approach is not ideally suited for thoroughly investigating adaptation compared to approaches such as whole‐genome resequencing (Lowry et al., 2017). Finally, identifying adaptive loci associated with environmental differences between putative Great Lakes and Boardman River groups would provide substantially more evidence to support the existence of unique genetic groups with differing life histories.

### 4.2 Ecological differences suggest ontogenetic habitat shifts

Our results suggest that the different putative genetic groups we detected in species such as rock bass and smallmouth bass may experience ontogenetic habitat shifts with movement between the BR and GL systems. Specifically, we hypothesize that, for these two species, juvenile fish from the GL group enter the lower Boardman River from Grand Traverse Bay for a period of time, likely to seek food and/or refuge. Additionally, some BR fish may also leave the river and enter the bay during parts of the year, as they are not captured consistently in the river below the dam throughout the year (R. Swanson, GLFC, personal communication). For rock bass and especially smallmouth bass, GL fish in the lower Boardman River were significantly smaller than the BR fish that they were mixing with. However, it is important to note that our length data must be interpreted with caution due to gear selectivity, variable capture dates and sample sizes, and a lack of age data; nevertheless, the trends we observed in these two species are unlikely to be statistical artifacts as they were highly consistent across the different groups we assessed. Data on variables like fish length and sampling date are available for many population genetics studies, but these data are rarely incorporated into conservation genomics research. We thus demonstrate the utility of incorporating ecological data to gain a clearer picture of fish life history and movement patterns, and we suggest that conservation genomic studies should explore incorporating this type of data more frequently.

Most fish species are known to demonstrate ontogenetic niche shifts, switching to different food sources or habitats during different life‐history stages, and movement to nearshore, wetland, or tributary habitats represent a common ontogenetic shift for growing fishes (Persson & Crowder, 1998; Werner & Gilliam, 1984). In Lake Michigan, river mouths and tributaries are unique ecosystems that harbor diverse and variable fish assemblages (Janetski & Ruetz, 2015; Larson et al., 2013). These systems are generally characterized by relatively warmer temperatures, higher productivity and turbidity, and more macrophyte cover compared to the larger lake to which they are connected (Höök et al., 2007; Larson et al., 2013). Consequently, these habitats are important nursery and refuge areas for juvenile fish (Altenritter et al., 2013; Brazner & Beals, 1997; Madenjian et al., 2018). In our study, evidence for an ontogenetic habitat shift of young GL fish into the lower Boardman River was strongest for smallmouth bass. While this species is often considered relatively sedentary, Humston et al. (2017) found that young smallmouth bass exhibited a high degree movement between river systems and tributaries, with some age‐0 fish traveling at least several km from their natal site and suggested that differences in lake and river habitats could be driving this dispersal. Similar variations in life‐history strategies have recently been uncovered in some of our other study species as well. Chorak et al. (2019) and Senegal et al. (2020) both found that in eastern Lake Michigan, yellow perch exhibited multiple life‐history variations, in which some yellow perch were Lake Michigan residents, some were DRM lake residents, and some were Lake Michigan fish that temporarily moved into DRM lakes during the fall. It is therefore possible that prior to dam construction, GL juvenile fish from some or all of our species not only entered the lower Boardman River, as we observed in our study, but also traveled upstream of the current dam site and utilized habitat in Boardman Lake, which is a natural DRM lake, and perhaps further upstream as well. Again, it is important to note that our hypotheses about ontogenetic shifts and differential habitat use by distinct genetic groups could be strengthened by additional sampling. Sampling in the Boardman River watershed and Grand Traverse Bay consistently throughout the year, sampling across multiple years, and collecting length and age data would provide more insight into the life histories and seasonal use of different habitats during various life stages for each genetic group.

### 4.3 Conservation/management implications and conclusions

We hypothesize that the genetic structure of our five study species in the Boardman River has likely been influenced more by historical genetic divergence caused by multiple natural and anthropogenic factors than solely by fragmentation due to the Union Street Dam. Specifically, our data suggest that complex and interacting processes have shaped the genetic structure of fish in our study system, ranging from differentiation caused by stocking (walleye), to maintenance of unique genetic groups despite mixing below the dam (smallmouth bass, rock bass), to subtle differentiation with potentially high degrees of admixture (white sucker and yellow perch). It is important to note that even if the Union Street Dam is not the primary driver of these patterns of population structure, it is likely still influencing them, as it has hindered the ability of fish to migrate above the dam into the Boardman River watershed.

Without our genetic assessment, the presence of the cryptic genetic diversity that exists across small spatial scales in the lower Boardman River would have continued undetected. Our findings highlight the fact that fish from genetically distinct groups may sometimes overlap spatiotemporally and may also have distinctive life histories with unique population dynamics and habitat requirements during different life stages. These multiple life histories are likely important components of a robust portfolio of within‐species diversity that promotes population stability and resilience in the face of environmental stochasticity (Schindler et al., 2010).

Even if river fragmentation does not result in population declines or drastic genetic impacts as some studies have observed, the restoration of connectivity between lake and tributary habitat is still essential for conservation and restoration of fish populations. Connectivity between Lake Michigan, Boardman Lake, and the entire Boardman watershed was likely historically important for various components of the life histories of all our study species. Species that migrate from the Great Lakes into tributaries to spawn like white sucker and walleye are more obviously impacted by barriers, but our research suggests that species like rock bass and smallmouth bass, which may not exhibit similarly distinct spawning runs over long distances, may also be negatively impacted by fragmentation. Even if their utilization of river habitat is less obvious, populations of these species in Lake Michigan likely rely upon tributary and DRM habitats, especially as juveniles, for feeding and refuge. The role of tributary and DRM habitat in Great Lakes fishery production has been underappreciated, but studies like ours are illuminating the importance of protecting these unique ecosystems feeding into the Great Lakes, which play a vital role in fish recruitment, growth, and reproduction. Based on our results, we suggest that fisheries managers in the Great Lakes and beyond adopt a more holistic viewpoint of fish populations and the habitats they occupy that considers the existence of unique and partially sympatric genetic groups as well as the importance of habitat connectivity across multiple life stages. For example, fisheries managers could conduct studies to understand the full range of habitats that species and genetic groups occupy throughout their lifetimes and attempt to protect all habitats rather than just spawning sites or areas where fish are harvested.

In conclusion, our study combined genetic and ecological data to illuminate cryptic population diversity and heterogeneous patterns of differentiation that could have major implications for how fish populations in the Great Lakes are managed. Specifically, identifying cryptic genetic groups will allow managers to design strategies that ensure these groups are not overharvested and conserve the habitats that they rely on, thereby preserving a robust portfolio of diversity that will lead to more sustainable fisheries and more consistent ecosystem services. Additionally, our workflow which included ecological data, genetic data, and simulations can be applied to other systems to investigate the relative importance of dams and other factors in shaping population structure and how it may vary among species. In conjunction with traditional survey methods, genetics has the power to elucidate cryptic and underappreciated diversity. We suggest that resource managers seek to incorporate genetic analysis into their toolbox more frequently to better understand and conserve important habitats and populations.

## 5 CONFLICT OF INTEREST

The authors declare no conflict of interest.

## Supporting information

Fig S1

Fig S2

Fig S3

Fig S4

Table S1

Table S2

Table S3

## Data Availability

Raw sequence reads used in this research along are archived with NCBI under BioProject ID PRJNA735793 http://www.ncbi.nlm.nih.gov/bioproject/735793. Scripts used for bioinformatics are available upon request.
