# Supplementary figures and images for: It’s complicated: Heterogeneous patterns of genetic structure in five fish species from a fragmented river suggest multiple processes can drive differentiation
Source: Evol Appl. 2021 Jun 29;14(8):2079–97. doi: 10.1111/eva.13268 (PMC8372089; doi:10.1111/eva.13268)

# Rock bass

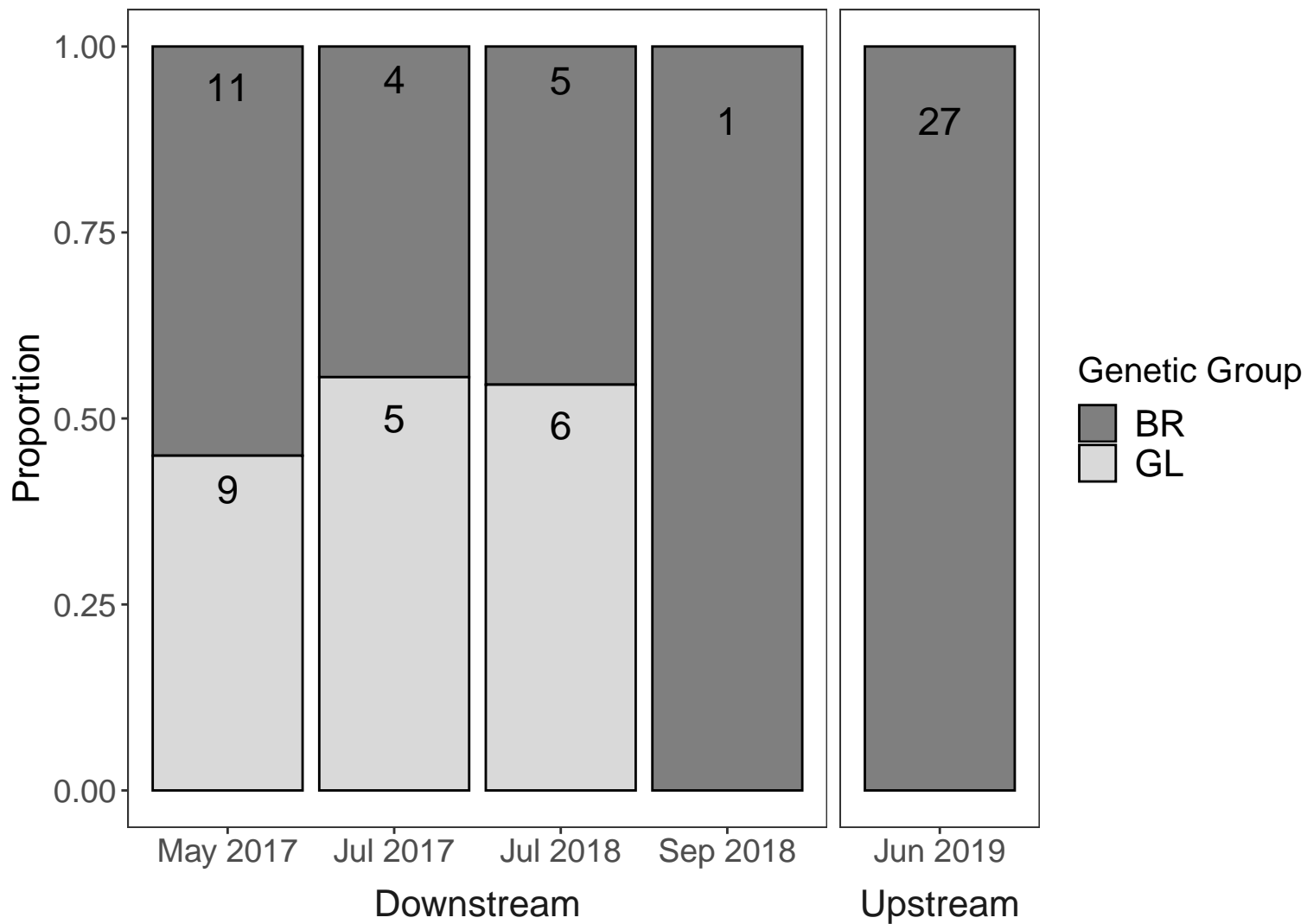

# White Sucker

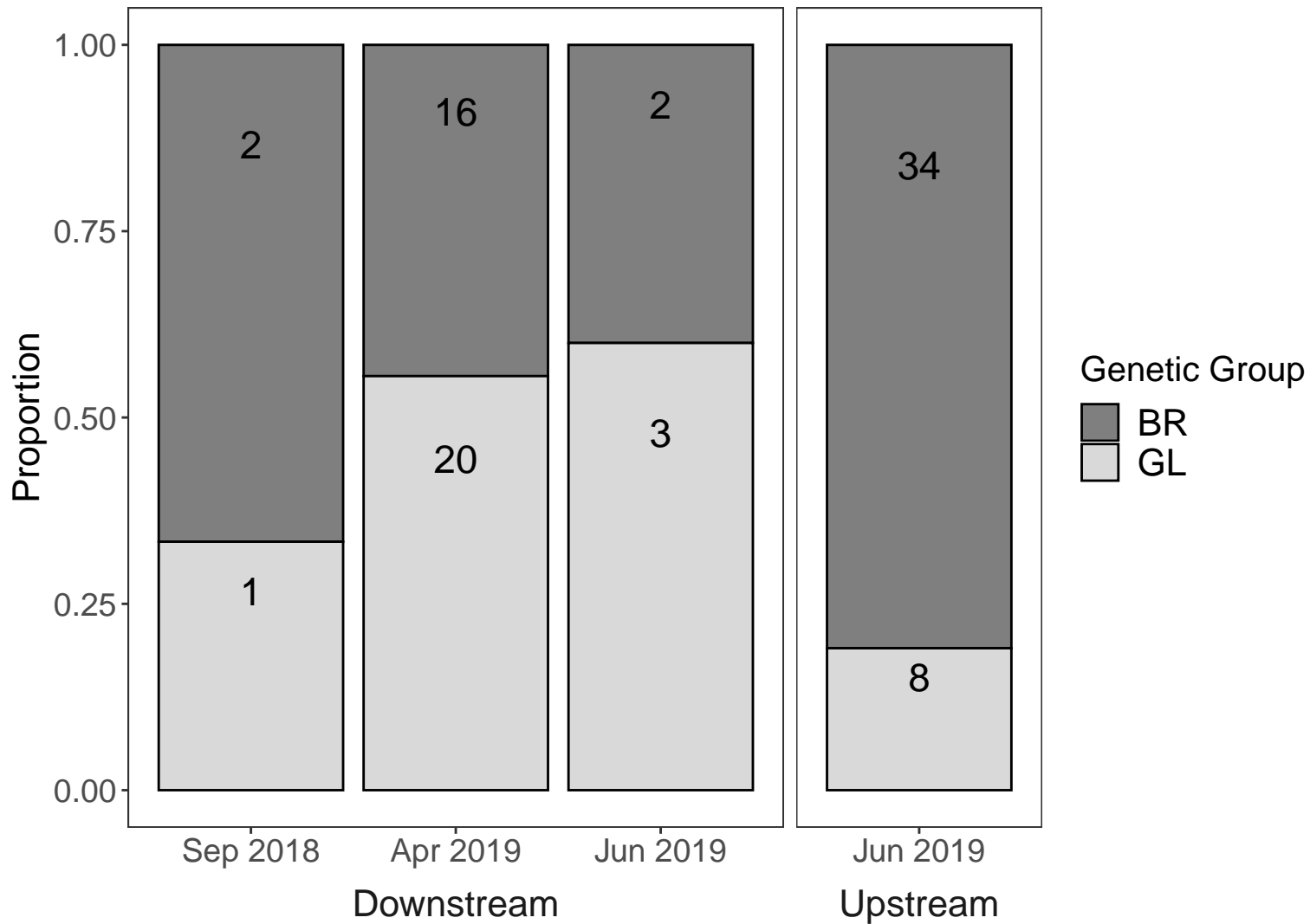

# Smallmouth bass

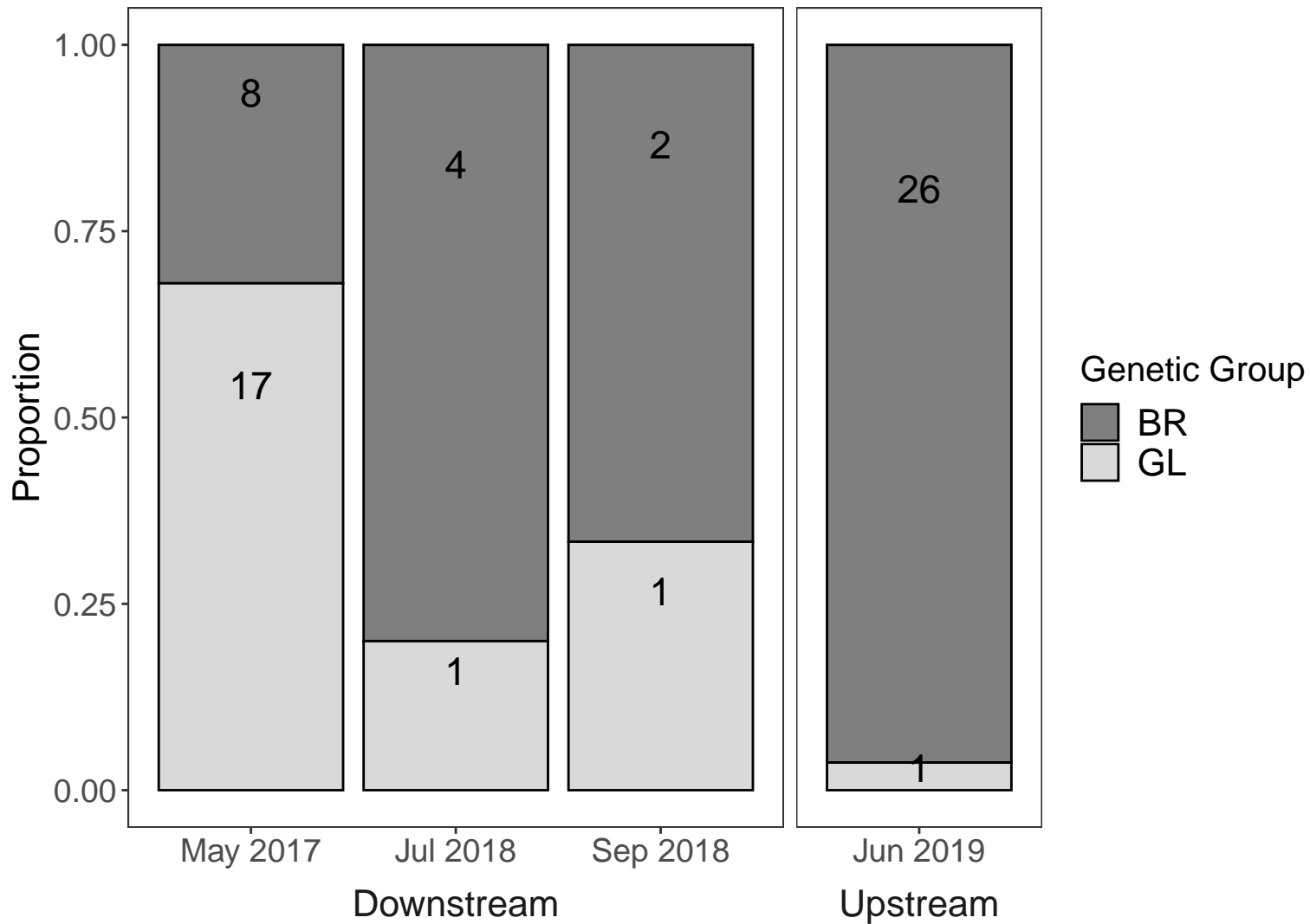

# Yellow perch

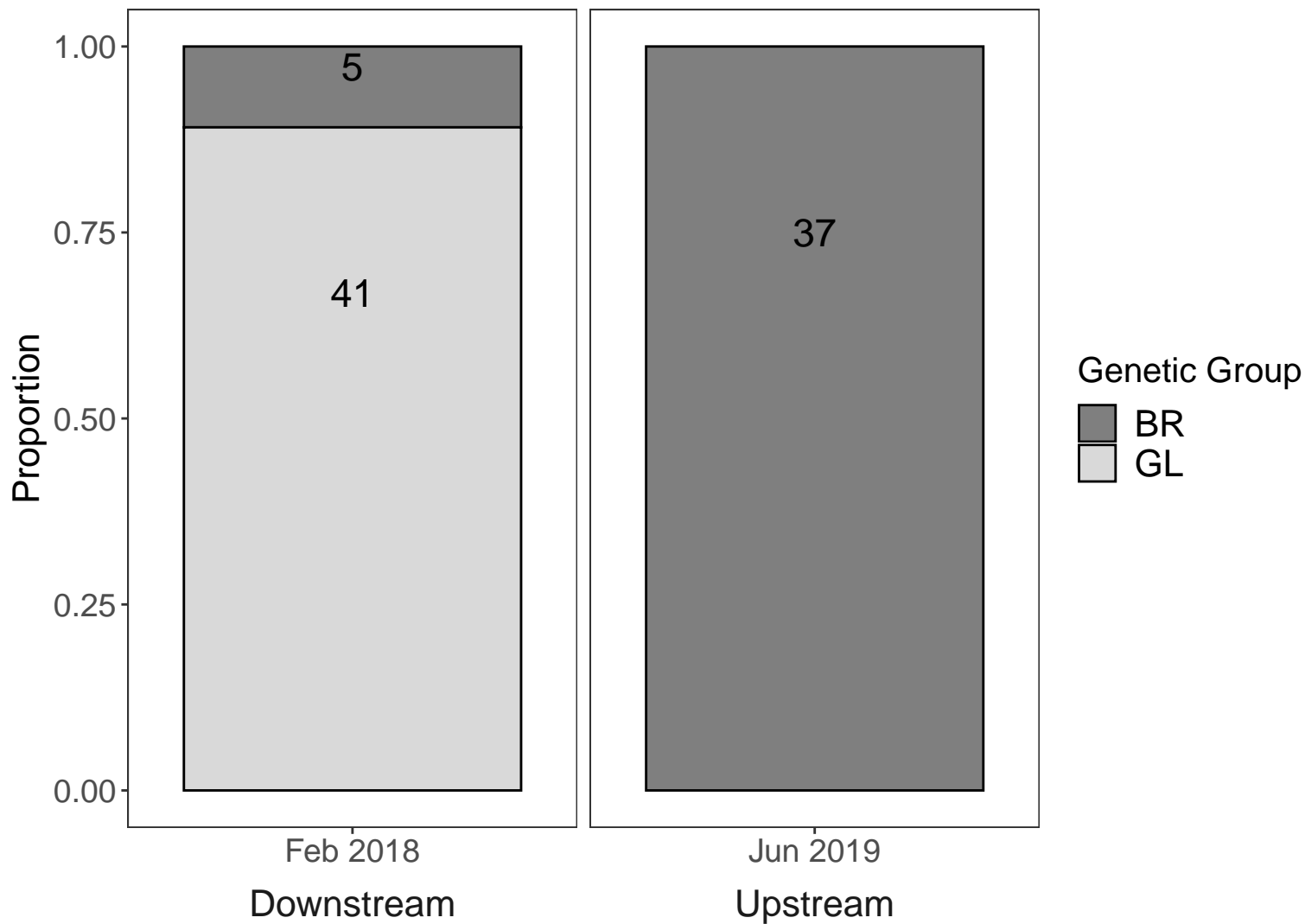

# Walleye

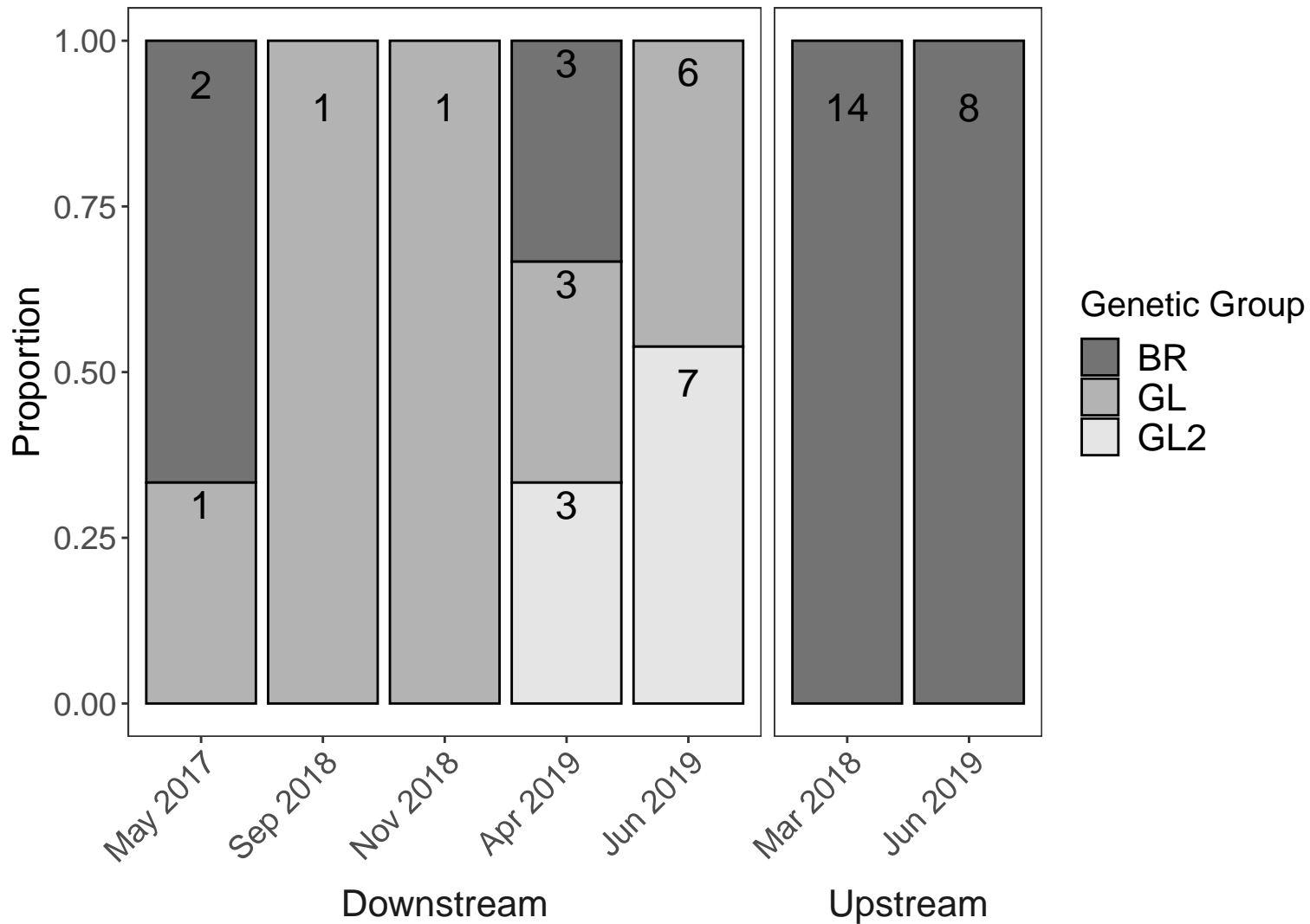

Supplement: Supplementary file 1 — Fig S1 [file EVA-14-2079-s003.pdf]

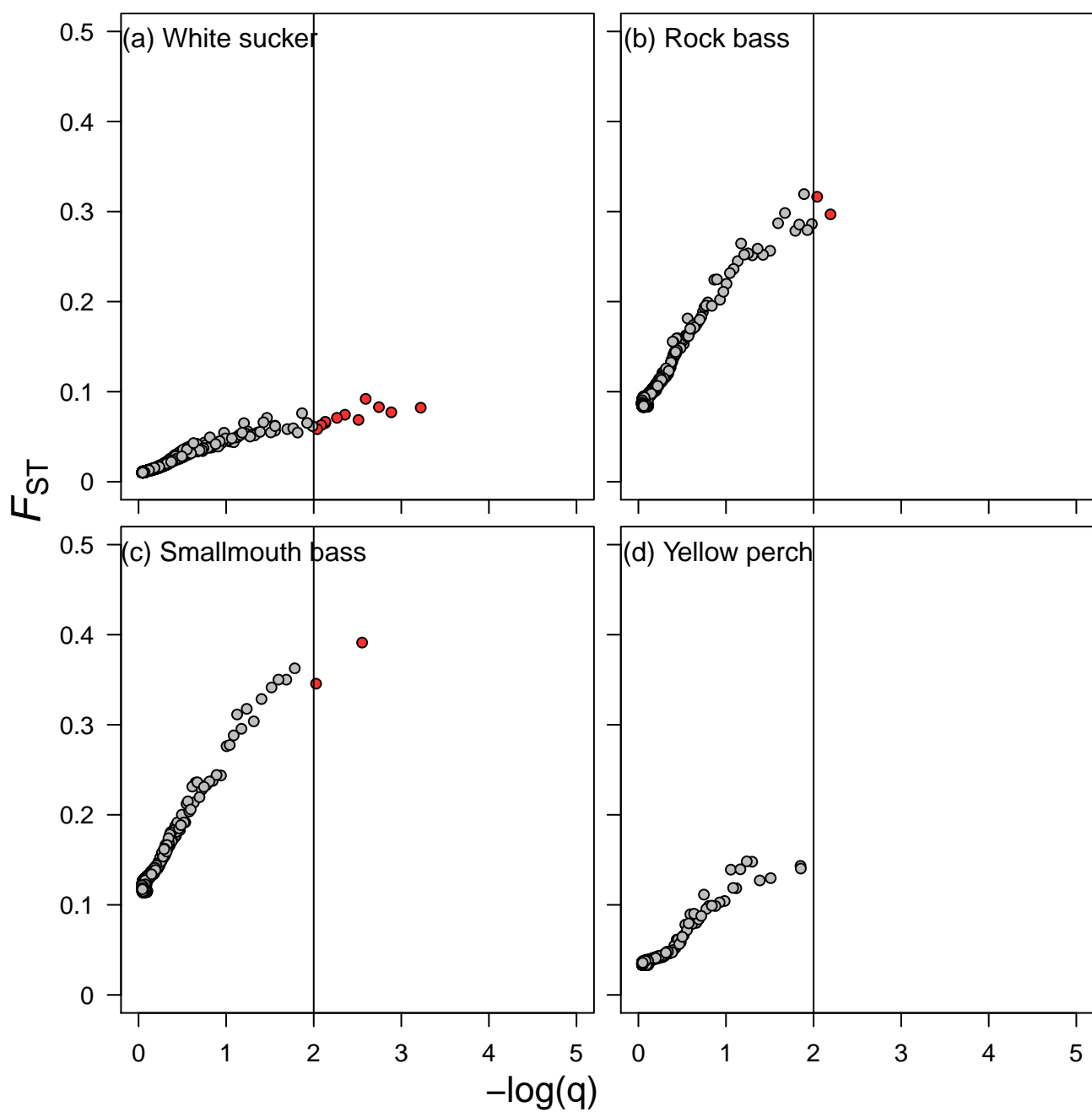

Supplement: Supplementary file 3 — Fig S3 [file EVA-14-2079-s002.pdf]

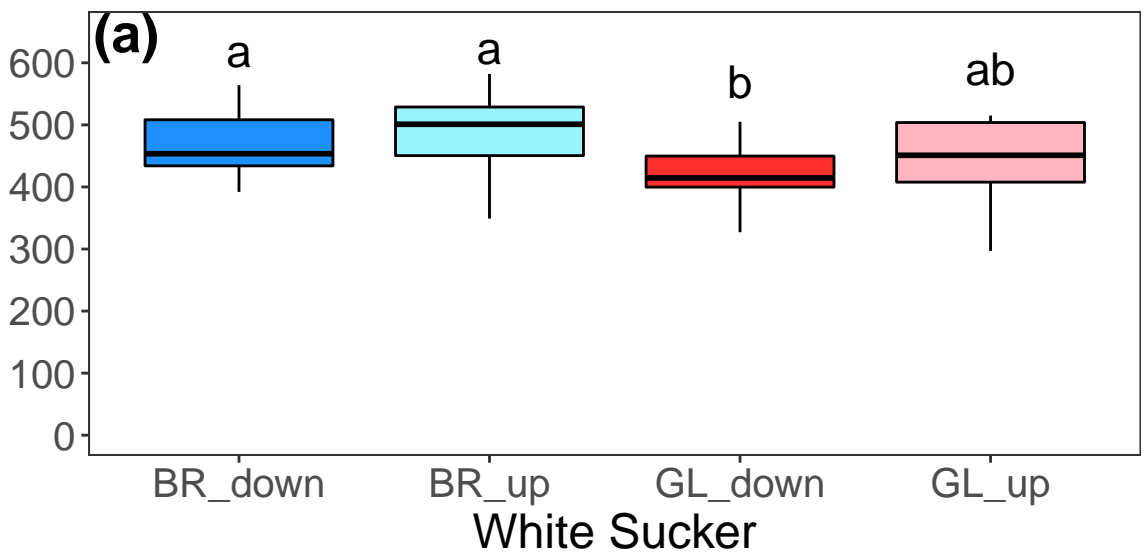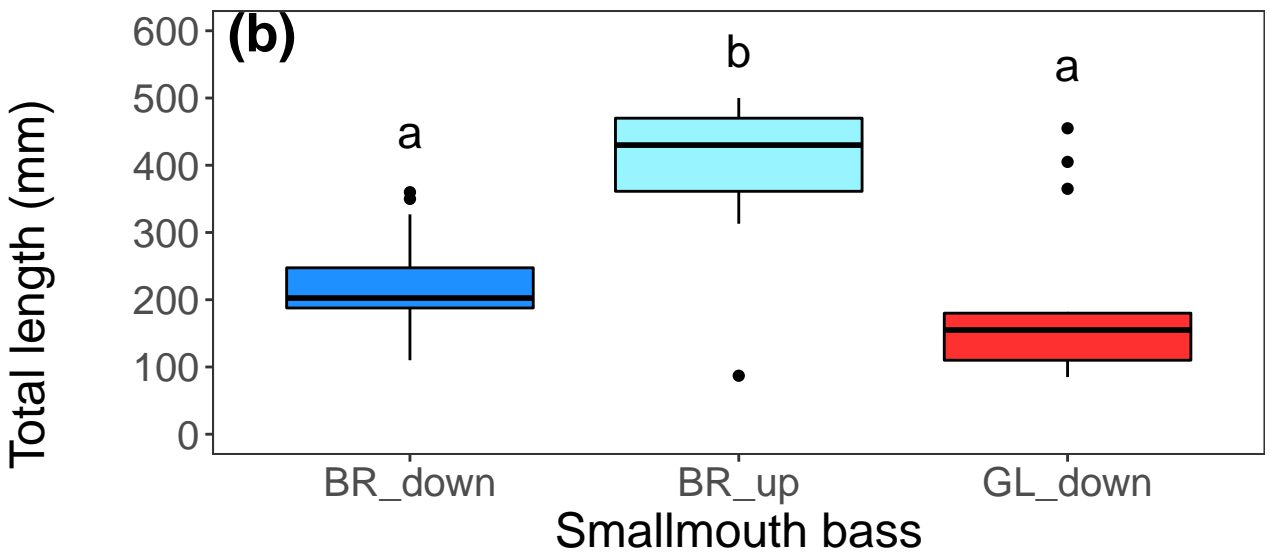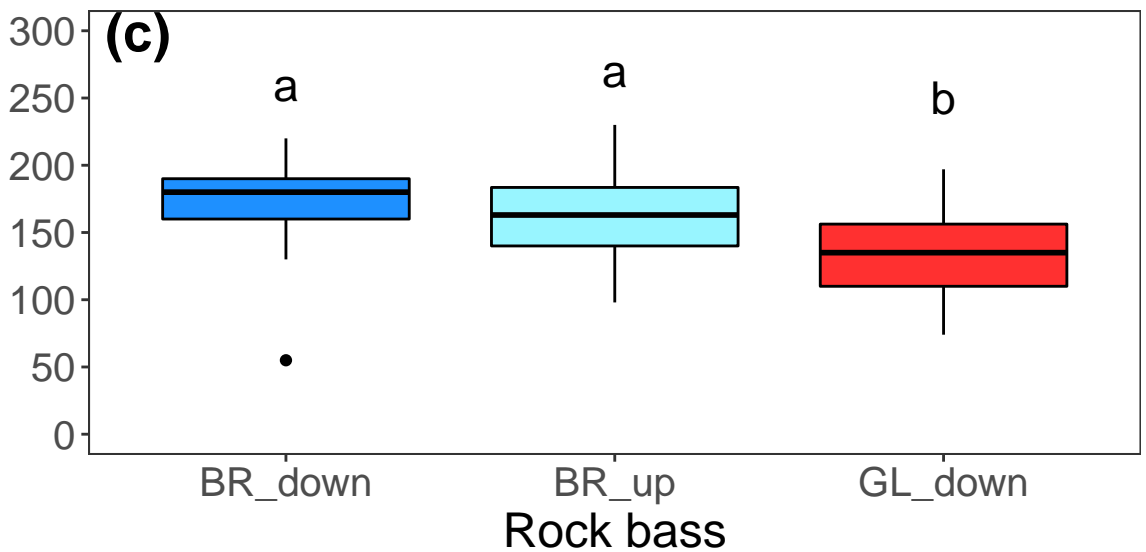

Supplement: Supplementary file 4 — Fig S4 [file EVA-14-2079-s006.pdf]
