# Supplementary material for: It’s complicated: Heterogeneous patterns of genetic structure in five fish species from a fragmented river suggest multiple processes can drive differentiation
Source: Evol Appl. 2021 Jun 29;14(8):2079–97. doi: 10.1111/eva.13268 (PMC8372089; doi:10.1111/eva.13268)
Supplement: Supplementary file 2 — Fig S2 [file EVA-14-2079-s001.pdf]

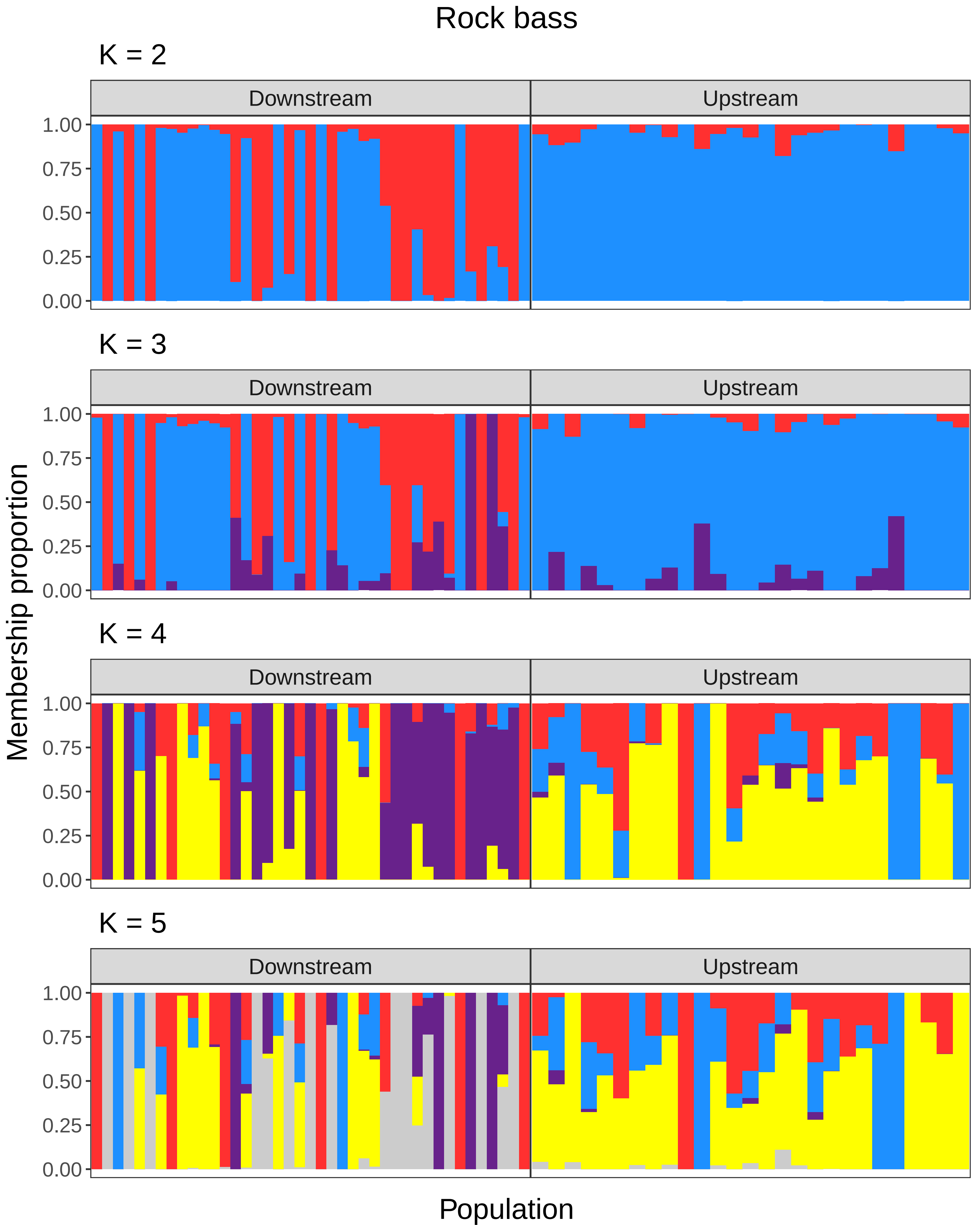

Rock bass
K = 2
K = 3
K = 4
K = 5
Downstream
Upstream
Membership proportion
1.00
0.75
0.50
0.25
0.00
Population

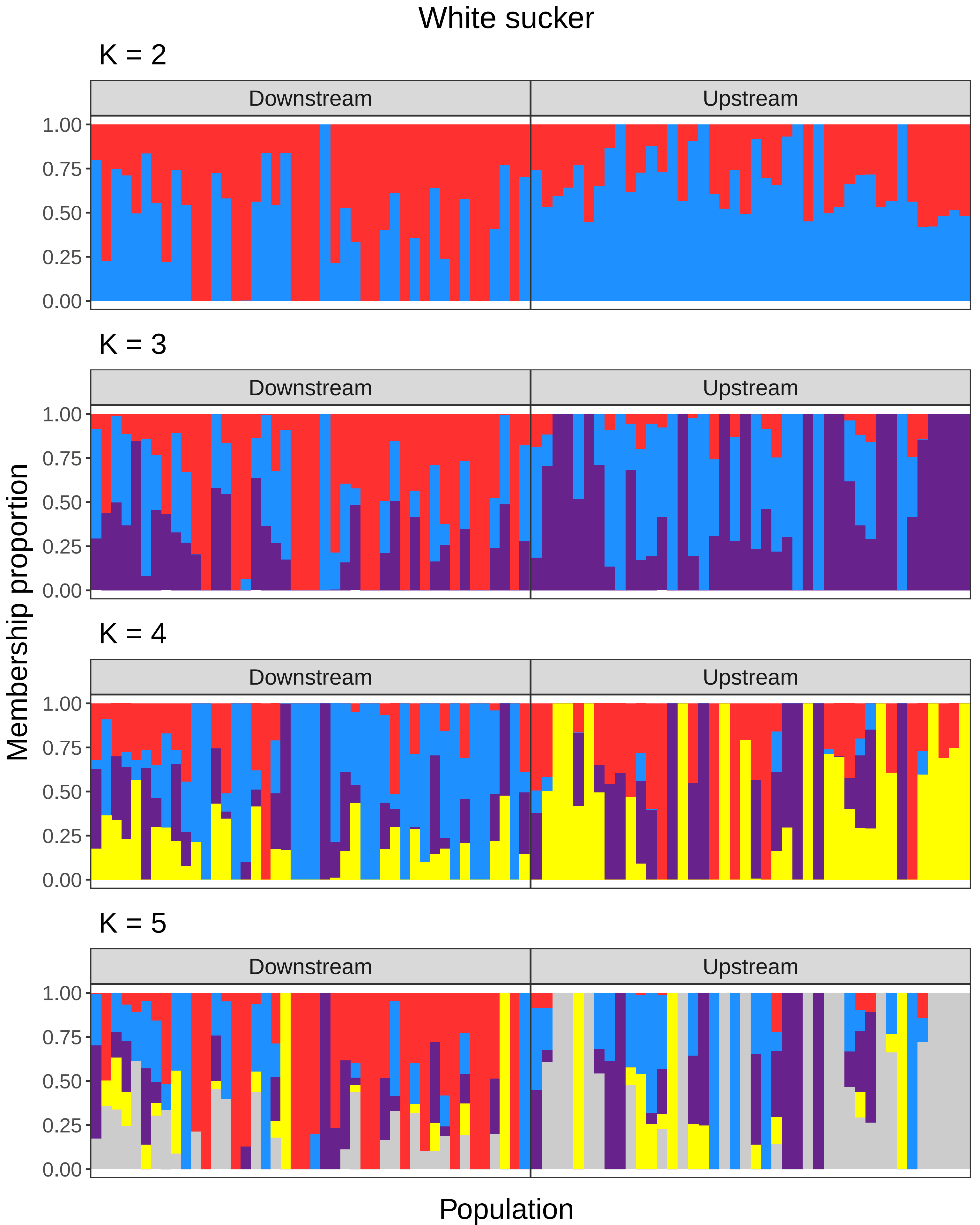
White sucker
K = 2
Downstream
Upstream
K = 3
Downstream
Upstream
K = 4
Downstream
Upstream
K = 5
Downstream
Upstream
1.00
0.75
0.50
0.25
0.00
Membership proportion
Population

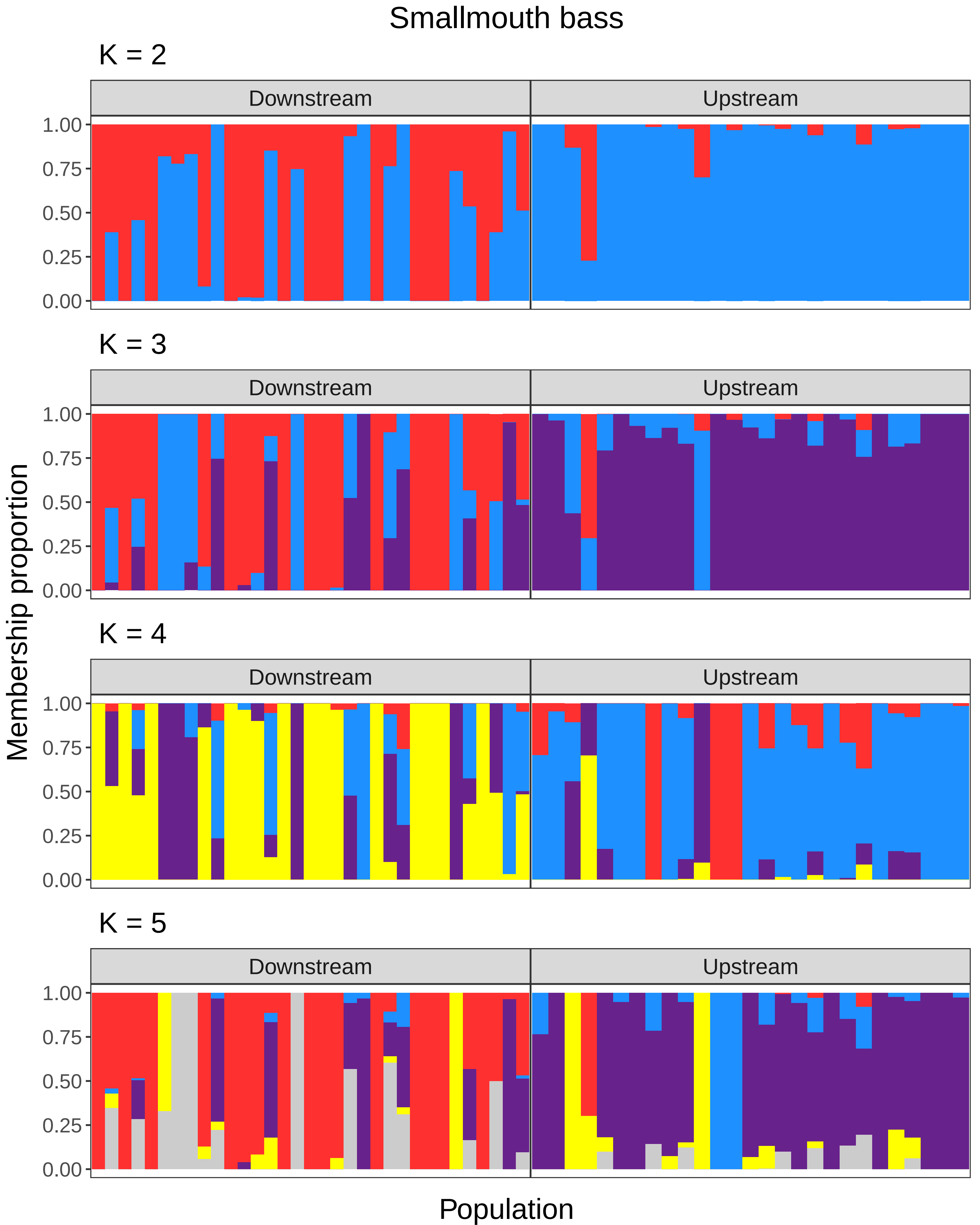
Smallmouth bass
K = 2
K = 3
K = 4
K = 5
Downstream
Upstream
1.00
0.75
0.50
0.25
0.00
Membership proportion
Population

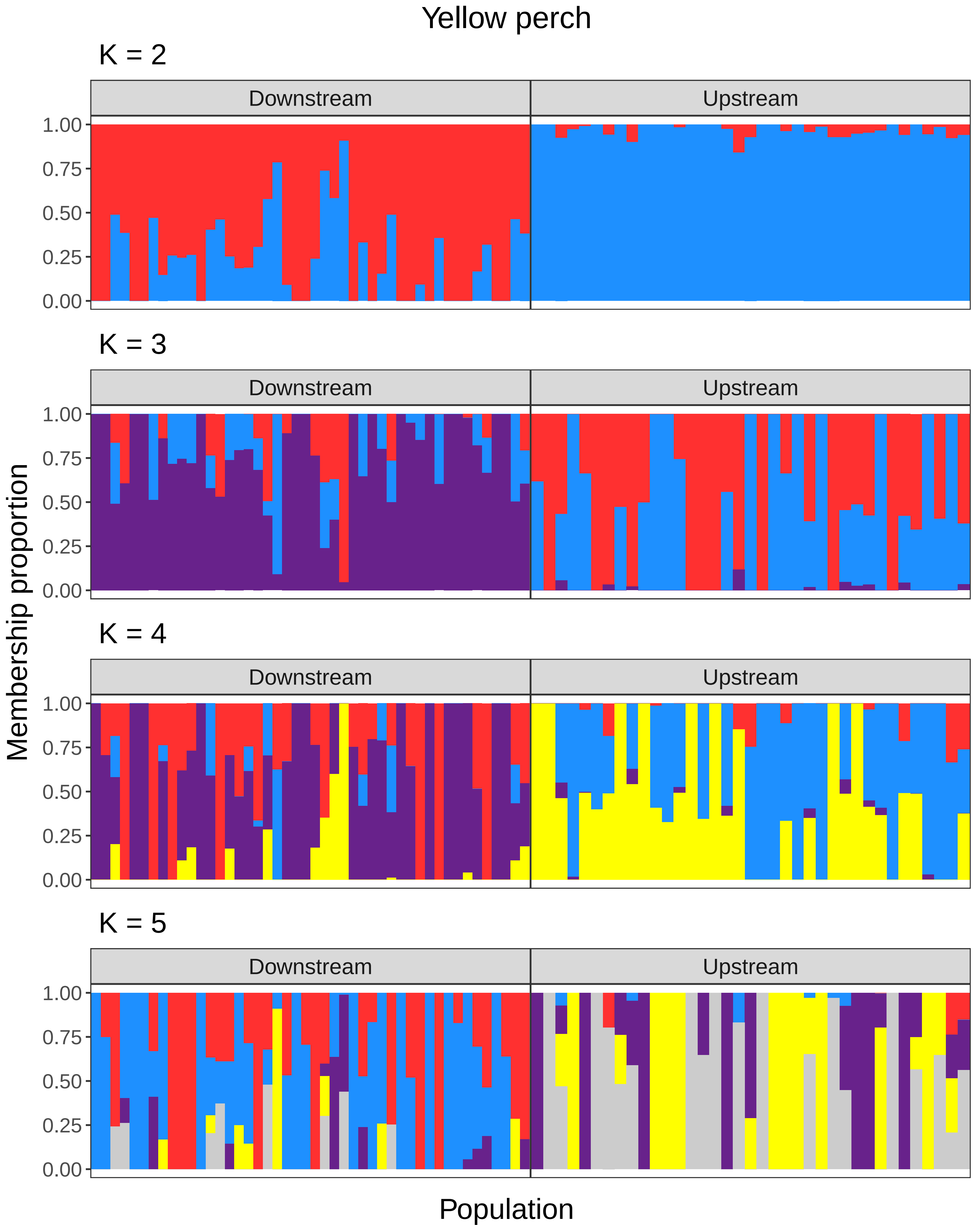

Yellow perch
K = 2
Downstream
Upstream
K = 3
Downstream
Upstream
K = 4
Downstream
Upstream
K = 5
Downstream
Upstream
1.00
0.75
0.50
0.25
0.00
Membership proportion
Population

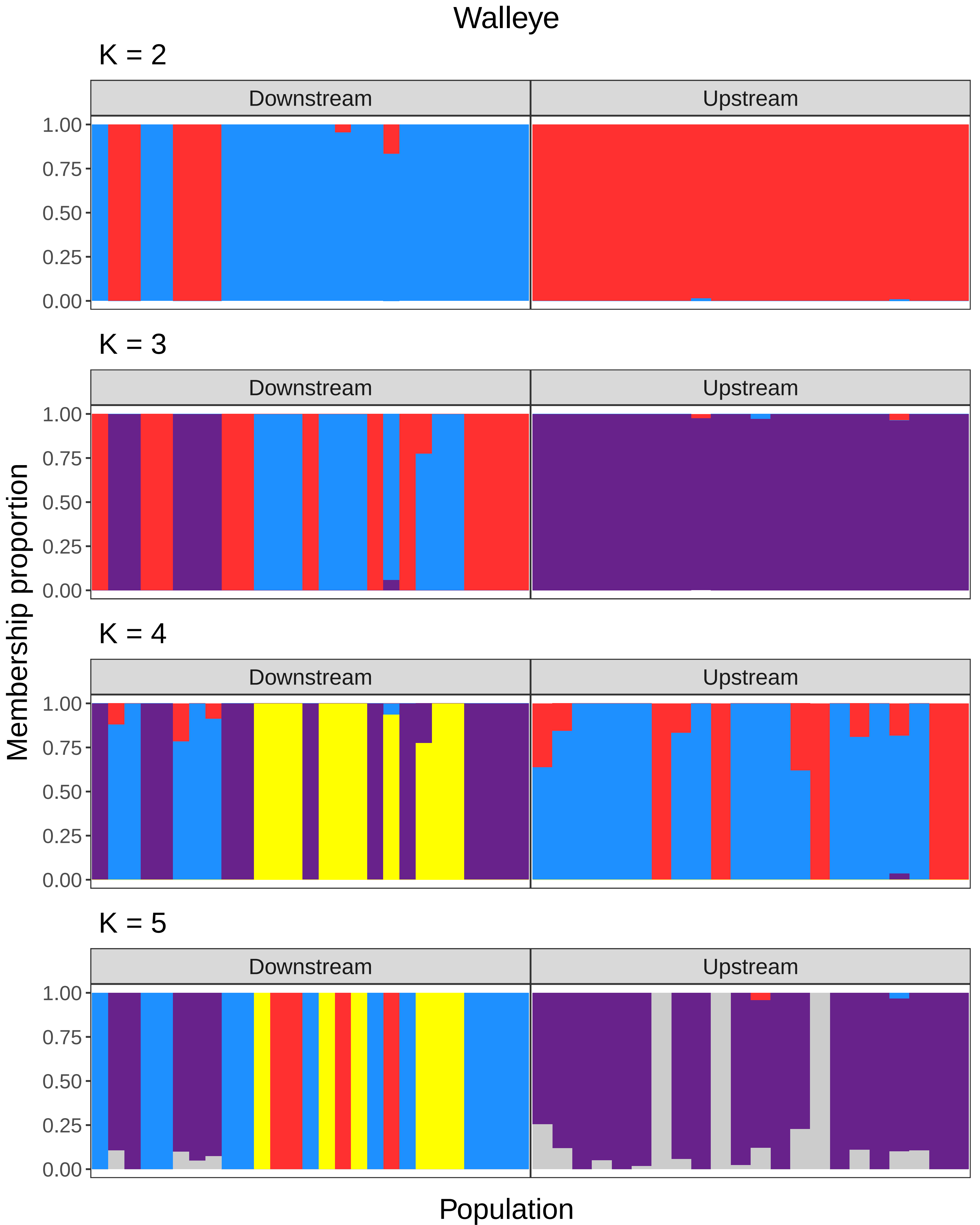
Walleye
K = 2
K = 3
K = 4
K = 5
Downstream
Upstream
1.00
0.75
0.50
0.25
0.00
Membership proportion
Population
